# *N*‐(Anilinoethyl)amide Melatonergic Ligands with Improved Water Solubility and Metabolic Stability

**DOI:** 10.1002/cmdc.202100405

**Published:** 2021-07-26

**Authors:** Francesca Ferlenghi, Michele Mari, Gabriella Gobbi, Gian Marco Elisi, Marco Mor, Silvia Rivara, Federica Vacondio, Silvia Bartolucci, Annalida Bedini, Fabiola Fanini, Gilberto Spadoni

**Affiliations:** ^1^ Dipartimento di Scienze degli Alimenti e del Farmaco Università degli Studi di Parma Parco Area delle Scienze 27/A 43124 Parma Italy; ^2^ Dipartimento di Scienze Biomolecolari Università degli Studi di Urbino Carlo Bo Piazza Rinascimento 6 61029 Urbino Italy; ^3^ Department of Psychiatry McGill University Montreal QC H3A1A1 Canada; ^4^ McGill University Health Center Montreal QC H31A1 Canada; ^5^ Microbiome Research Hub University of Parma 43124 Parma Italy

**Keywords:** drug-design, lipophilicity, melatonin receptors, metabolism, pharmacokinetics

## Abstract

The MT_2_‐selective melatonin receptor ligand UCM765 (*N*‐(2‐((3‐methoxyphenyl)(phenyl)amino)ethyl)acetamide), showed interesting sleep inducing, analgesic and anxiolytic properties in rodents, but suffers from low water solubility and modest metabolic stability. To overcome these limitations, different strategies were investigated, including modification of metabolically liable sites, introduction of hydrophilic substituents and design of more basic derivatives. Thermodynamic solubility, microsomal stability and lipophilicity of new compounds were experimentally evaluated, together with their MT_1_ and MT_2_ binding affinities. Introduction of a *m*‐hydroxymethyl substituent on the phenyl ring of UCM765 and replacement of the replacement of the *N,N*‐diphenyl‐amino scaffold with a *N‐*methyl‐*N*‐phenyl‐amino one led to highly soluble compounds with good microsomal stability and receptor binding affinity. Docking studies into the receptor crystal structure provided a rationale for their binding affinity. Pharmacokinetic characterization in rats highlighted higher plasma concentrations for the N‐methyl‐N‐phenyl‐amino derivative, consistent with its improved microsomal stability and makes this compound worthy of consideration for further pharmacological investigation.

## Graphical abstract

**A taste for stability**: Compound **19** is a potent melatonin receptor agonist obtained from the structural optimization of UCM765, an MT_2_‐selective partial agonist active in vivo, but characterized by poor physicochemical and pharmacokinetic properties. Insertion of a bromine atom on the phenyl‐methylamino scaffold led to a more soluble derivative endowed with higher microsomal stability.

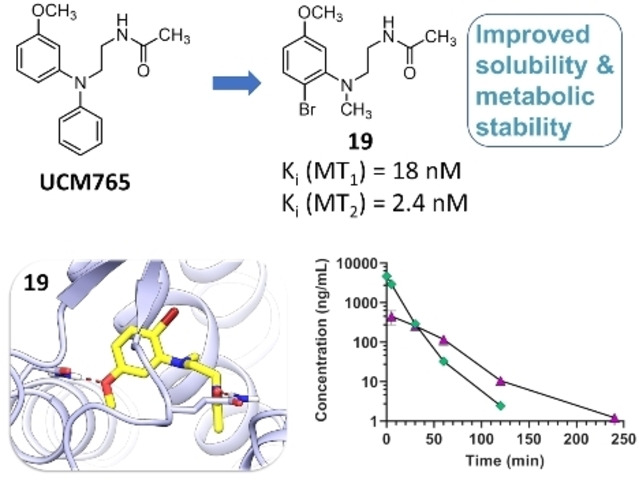

## Introduction

**Figure 1:**
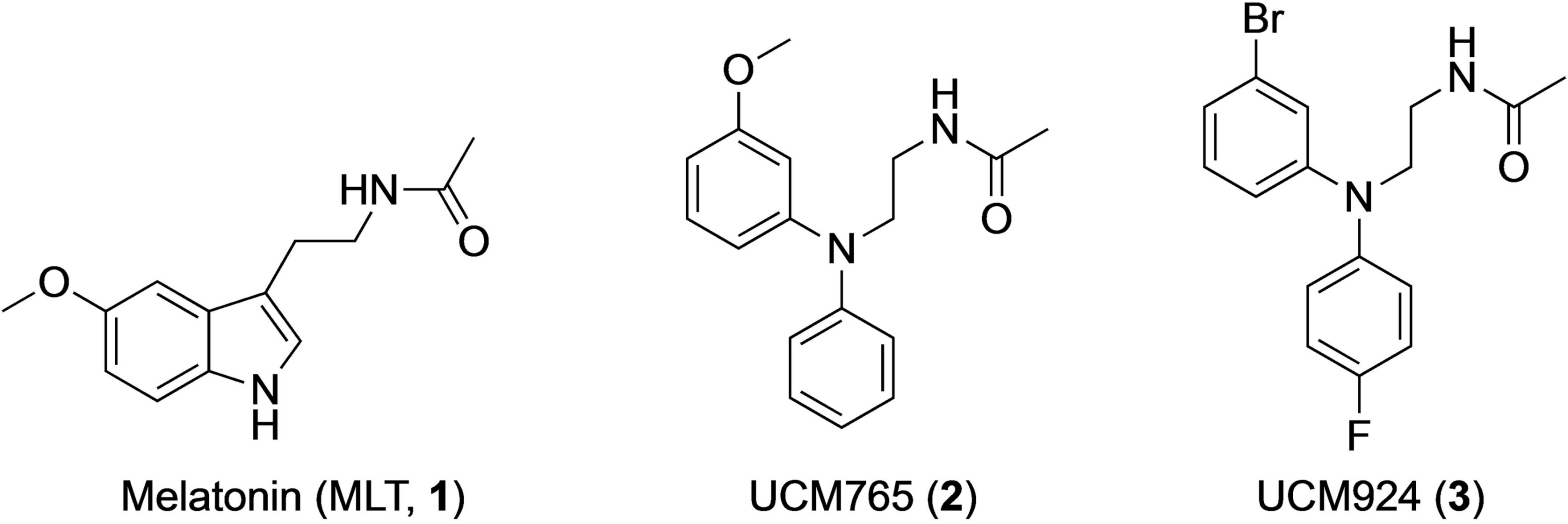
Melatonin and representative N‐(anilinoethyl)amide melatonin receptor ligands.

Melatonin (*N*‐acetyl‐5‐methoxytryptamine, MLT, **1**, Figure 1) is a neurohormone mainly produced by the pineal gland in a circadian rhythmic fashion with the highest levels, in both diurnal and nocturnal mammals, during the night dark phase.^[1]^ Other more local sources of MLT synthesis have been described including the retina,^[2]^ which also produces MLT in a circadian manner, and various human cells (leucocytes,^[3]^ lymphocytes,^[4]^ phagocytes,^[5]^ and placental trophoblasts,^[6]^) which synthesize MLT in a noncircadian manner, and do not contribute to the plasma hormone levels.

The circadian pattern of MLT secretion, coupled with the localization of specific MLT binding sites in the brain region associated with the “biological clock” has led to considerable interest in its potential in treating disordered circadian rhythms. In many countries, MLT is commonly used as a safer alternative to benzodiazepines and other sleeping aids for the treatment of insomnia, and to alleviate jet lag.

Apart the above cited sleep‐inducing and chronobiotic properties, several other functions have been attributed to MLT at physiological concentrations and even more when considering supraphysiological levels, including modulation of the activity of the immune system, regulation of the cardiovascular functions, control of mood and behavior, glucose homeostasis, hormone secretion and pain perception.^[7]^ Other effects of MLT described in the literature include its neuroprotective, anti‐inflammatory, retinal, antioxidant and anticancer properties.^[8]^ Despite the extensive number of studies reporting a multitude of MLT effects, the scientific evidence supporting the potential benefits of MLT assumption in humans remains controversial.^[9]^ Several molecular targets have been proposed for MLT since its discovery. Among them, the best‐characterized molecular targets are the two G protein‐coupled receptors MT_1_ and MT_2_. In mammals, these receptors are expressed in the brain, particularly in the *pars tuberalis* and hypothalamus, and in the retina, but traces of their expression can be found in some other organs.^[10]^ In the recent years, several studies have attempted to better characterize the localization and the function of these receptors, and it was found that they have a specific localization in the brain,^[11]^ and specific physiological functions,^[12]^ sometime also opposite.[^13^, ^14^]

A third melatonin‐related receptor (GPR50) has been identified in mammals that interacts with MT_1_ to negatively regulate its function. Whereas MT_1_ and MT_2_ receptors bind melatonin with sub‐nanomolar affinity and are the target of some currently prescribed drugs for circadian disorders, insomnia, and major depression, GPR50 does not bind MLT, despite its 50 % sequence identity with MT_1_ and MT_2_ melatonin receptors.^[15]^ The pharmacological characterization has been recently complemented by crystallization studies of human MT_1_ and MT_2_ receptors in complex with several melatonin analogs.[^15^, ^16^] Besides GPCRs, another low‐affinity MLT binding site, termed MT_3_, has been characterized as a melatonin‐sensitive form of the human enzyme NRH‐quinone oxidoreductase 2 (NQO2).^[18]^ Although research on the melatonergic system has expanded in the last years, more remains to be done to fully understand the unique structural features of each MLT receptor subtype and their function. Efforts to define the therapeutic potential of MT_1_ and MT_2_ receptors have led to the design and synthesis of several ligands.^[19]^ These compounds, which activities span from full agonist, to partial agonist, antagonist and inverse agonist, belong to structurally different classes that range from simple indole derivatives and their bioisosteres, to ring opened derivatives^[20]^ and conformationally restricted ligands.^[21]^ Among these, one of the most interesting and versatile classes was that of the N‐anilinoethylamides, which provided high affinity nonselective full agonists, MT_2_ selective partial agonists or very selective MT_2_ antagonists, depending on the size of the substituent on the aniline nitrogen.^[22]^ Two representative ligands of this class are the MT_2_‐selective partial agonist UCM765 (*N*‐{2‐[(3‐methoxyphenyl)phenylamino]ethyl}acetamide, **2** Figure 1), and its metabolically more stable congener UCM924 (*N*‐{2‐[(3‐bromophenyl)‐(4‐fluorophenyl)amino]ethyl}acetamide),^[23]^ **3**, Figure 1) that evidenced interesting sleep inducing,^[24]^ antinociceptive^[25]^ and anxiolytic^[26]^ properties in rodents. However, the suboptimal physicochemical properties of these compounds greatly limited their use in research and functional application, as they suffer from low aqueous solubility and modest microsomal stability. We herein investigated different strategies for the preparation of new N‐anilinoethylamide derivatives that could overcome these limitations, including modification of metabolically liable sites, such as the *m*‐methoxy and the N‐anilino substituents, or reduction of lipophilicity by replacement of the amide side chain or insertion of hydrophilic groups and design of more basic derivatives.

## Results and Discussion

### Chemistry

**Scheme 1:**
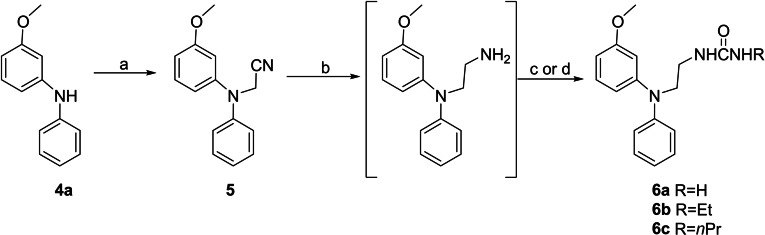
Synthesis of N‐[2‐(diphenylamino)ethyl]ureas **6 a**–**c**. *Reagents and conditions*: a) BrCH_2_CN, NaH, DMF, 100 °C, 24 h; b) H_2_, Raney‐Ni, NH_3_‐EtOH, THF, 60 °C, 6 h; c) KOCN, AcOH/THF/H_2_O, r.t., 4 h (for **6 a**); d) RNCO, DCM, r.t., 0.5 h (for **6 b‐c**).

The three urea melatonin receptor ligands **6 a**–**c** were prepared as described in the Scheme 1. The monosubstituted urea (**6 a**) was synthesized by reaction of the crude *N* ^1^‐3‐(methoxyphenyl)‐*N* ^1^‐phenylethane‐1,2‐diamine with potassium cyanate in acidic aqueous medium; disubstituted analogues (**6 b**–**c**) were obtained by treatment of the same ethane‐1,2‐diamine with ethyl‐ or *n*‐propyl isocyanate.

The crude *N* ^1^‐3‐(methoxyphenyl)‐*N* ^1^‐phenylethane‐1,2‐diamine was prepared following a previously reported procedure^[22]^ involving the N‐cyanoalkylation of 3‐methoxy‐*N*‐phenylaniline (**4 a**) with bromoacetonitrile in the presence of sodium hydride, and subsequent hydrogenation of the intermediate nitrile **5** with Raney Nickel, in the presence of 1 M NH_3_−EtOH.

**Scheme 2:**
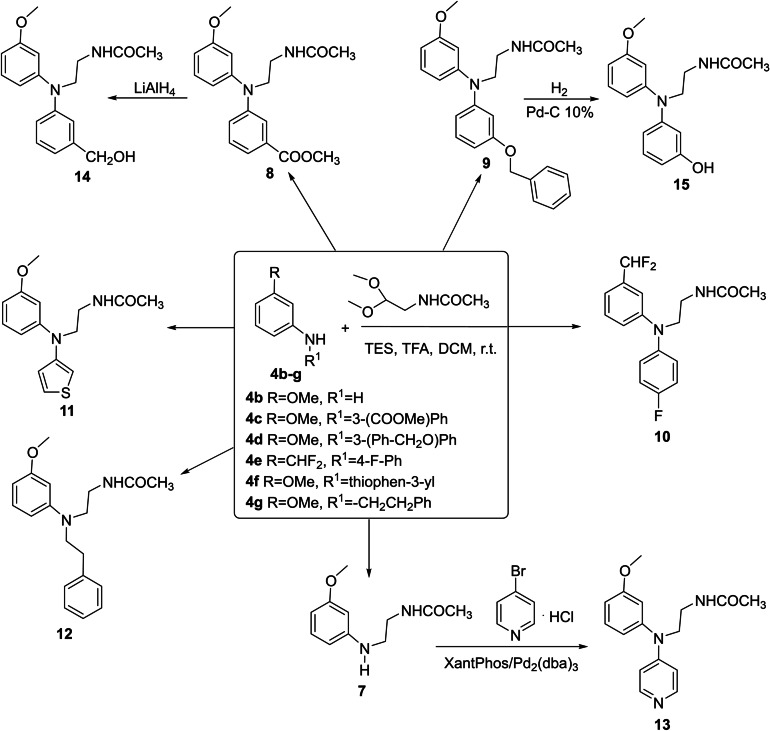
Synthesis of N‐[2‐(diarylamino)ethyl]amides **10**–**11**, **13**–**15** and N‐[2‐(N‐phenethylanilino)ethyl]acetamide **12**.

The (anilinoethyl)acetamido derivatives (**7**–**12**) were obtained by reductive N‐alkylation of the suitable aniline **4 b**–**g** with *N*‐(2,2‐dimethoxyethyl)acetamide in the presence of triethylsilane/trifluoroacetic acid (Scheme 2). The N‐pyridine aniline derivative **13** was synthesized by palladium‐catalyzed amination [Pd_2_(dba)_3_/XantPhos/*t‐*BuONa] of **7** with 4‐bromopyridine hydrochloride (Scheme 2). To prepare the 3‐(hydroxymethyl)phenyl derivative **14**, the corresponding methyl ester **8** was reduced with lithium aluminum hydride, while the 3‐hydroxyphenyl analog **15** was obtained by O‐debenzylation of compound **9**, using H_2_/Pd−C (Scheme 2).

**Scheme 3:**
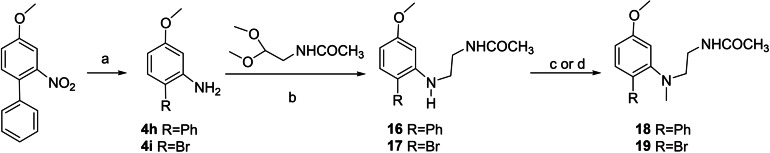
Synthesis of *N*‐[2‐(2‐substituted‐5‐methoxyanilino)ethyl]acetamides **18**–**19**. *Reagents and conditions*: a) H_2_, 10 % Pd−C, EtOAc/EtOH, r.t., 6 h; b) TES, TFA, DCM, r.t., 2 h; c) MeI, NaHCO_3_, MeOH, 50 °C, 24 h (for **18**); d) HCHO (37 % in aqueous solution), NaBH_3_CN, MeOH/AcOH, r.t., 1 h (for **19**).

The N‐methyl‐2‐substituted‐aniline derivatives **18**–**19** were prepared by reductive N‐alkylation of the corresponding 2‐substituted anilines **4 h**–**i** with *N*‐(2,2‐dimethoxyethyl)acetamide in the presence of triethylsilane/trifluoroacetic acid, followed by N‐methylation with MeI (**18**) or reductive amination with HCHO/NaBH_3_CN (**19**), as depicted in Scheme 3.

**Scheme 4:**
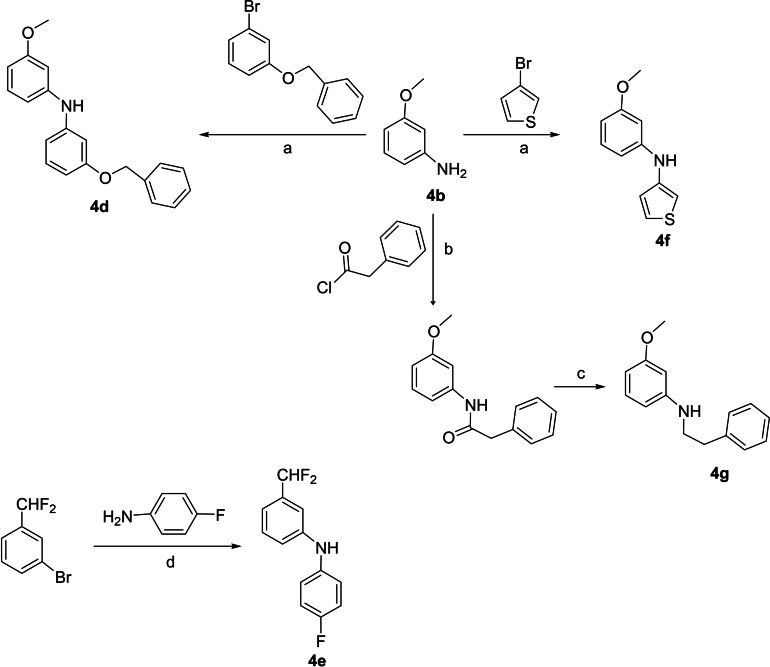
Synthesis of N,N‐diarylamines (**4 d**–**f**) and *N*‐phenethyl‐3‐methoxyaniline (**4 g**). *Reagents and conditions*: a) *t*‐BuONa, Pd_2_(dba)_3_, XantPhos for **4 d**, RuPhos for **4 f**, toluene, 100 °C, 18 h; b) TEA, DCM, r.t. 0.5 h; c) LiAlH_4_, THF, reflux, 1 h; d) BINAP, Pd(OAc)_2_, *t*‐BuOK, toluene, 100 °C, 3 h.

The key starting anilines are commercially available (**4 a**–**b**, **4 i**) or have been synthesized using known procedures for similar compounds as outlined in Scheme 4. Briefly, the 6*‐*phenyl‐*m*‐anisidine **4 h** was prepared by hydrogenation (H_2_/Pd−C) of 4‐methoxy‐2‐nitro‐1,1’‐biphenyl^[27]^ (Scheme 3).

The N‐arylanilines **4 d**–**f** were prepared by palladium‐catalyzed amination of the suitable aniline with 1‐(benzyloxy)‐3‐bromobenzene, 3‐bromothiophene or 3‐(difluoromethyl)bromobenzene (Scheme 4). The *N*‐phenethylaniline **4 g** was prepared by N‐acylation of 3‐methoxyaniline with phenylacetyl chloride and subsequent amide reduction by treatment with lithium aluminum hydride (Scheme 4).

### Biological evaluation and SAR discussion

MT_1_ and MT_2_ binding affinities of the new melatonergic ligands are reported in Table 1, with data for reference compounds UCM765 and UCM924 tested in the same experimental conditions. Different strategies were applied to improve the solubility and the metabolic stability of new compounds. We designed bioisosteres of the lead UCM765, with modifications of the amide side chain in urea derivatives **6 a**–**c**, of the methoxy group which was replaced with a difluoromethyl one (**10**) and of the N‐anilino substituent in compound **11** carrying a 3‐thienyl ring. Hydrophilic groups were inserted on the structure of UCM765, in the hydroxymethyl and phenol derivatives **14** and **15**, and the phenyl substituent was replaced with a pyridine in compound **13**.

**Table 1:** Binding affinity of melatonergic ligands at human MT_1_ and MT_2_ receptors.

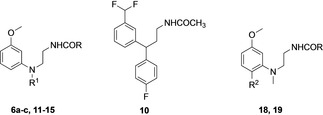					
Compd.	R	R^1^	R^2^	MT_1_ *K*_i_ [nM]^[a]^	MT_2_ *K*_i_ [nM]^[a]^
**2 (UCM765)**				1.6±0.14	0.29±0.10
**3 (UCM924)**				>100	1.4±0.08
**6 a**	NH_2_	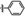		>100	10±0.13
**6 b**	NHEt	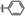		20±0.45	0.65±0.03
**6 c**	NH*n*Pr	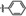		57±0.10	1.7±0.08
**10**				>100	35±0.35
**11**	CH_3_	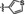		0.7±0.10	0.07±0.02
**12**	CH_3_	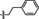		>100	2.1±0.15
**13**	CH_3_	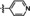		>100	>100
**14**	CH_3_	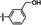		>100	3.5±0.05
**15**	CH_3_	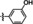		53±1.6	1.4±0.15
**18**			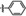	32±0.80	1.7±0.13
**19**			Br	18±0.70	2.4±0.10

[a] *K*_i_ values are calculated from IC_50_ values obtained from competition curves by the method of Cheng and Prusoff,^[28]^ and are the mean±standard deviation of three determinations.

**Figure 2:**
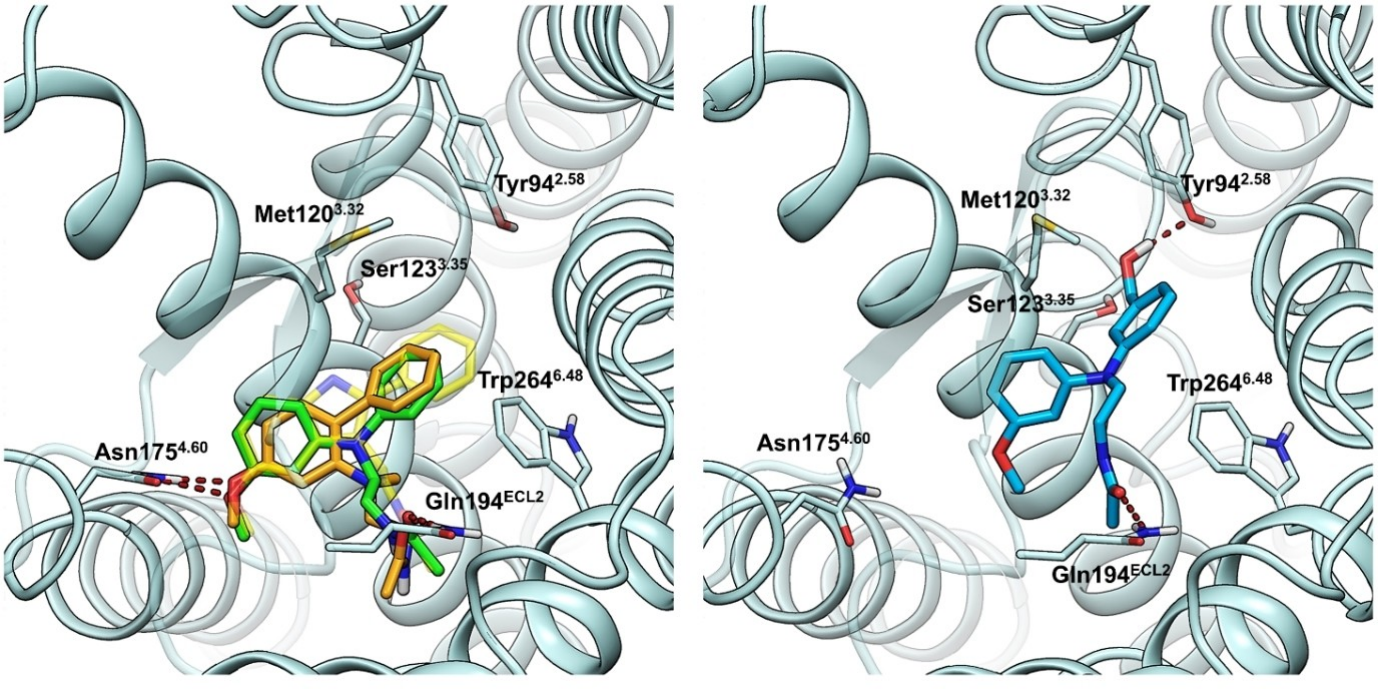
Ligand binding to the MT_2_ receptor. *Left*: Proposed binding mode for UCM765 (green carbons) and compound **18** (orange carbons) into the MT_2_ crystal structure (PDB: 6ME6), observed from the extracellular side of the receptor. Hydrogen bonds are shown as red dashed lines. Co‐crystalized 2‐phenylmelatonin is shown with shaded yellow carbons. Ballesteros‐Weinstein residue numbering^[32]^ is adopted in superscripts. *Right*: Representative snapshot of a molecular dynamics simulation of compound **14** in complex with the MT_2_ receptor. The ligand undertakes hydrogen bonds with Gln194 in ECL2 and Tyr94, while the interaction between the methoxy group and Asn175, proposed by docking studies, is lost.

A different approach was followed for the phenyl‐alkylamides **12**, **18** and **19**, more basic than the diphenylalkylamide derivatives. A phenethyl group was inserted on the aniline nitrogen in compound **12**, while for compounds **18** and **19** a change in the scaffold was obtained moving the *N*‐phenyl substituent of UCM765 on the methoxyphenyl ring, para to the methoxy group (**18**). In compound **19** phenyl substituent was replaced with the more metabolically stable bromine atom, while maintaining a similar lipophilicity to optimize receptor interaction. Urea derivatives showed reduced binding affinity, particularly at the MT_1_ receptor, with the most potent derivative **6 b** having MT_2_ binding affinity comparable to that of UCM765. The lower affinity of ureas compared to amide derivatives has already been reported for other melatonergic ligands, such as agomelatine and its structural analogs[^29^, ^30^] or tetrahydroquinolines.^[31]^ However, the lower lipophilic character could improve water solubility. Replacement of the metabolically labile methoxy substituent with a difluoromethyl group (**10**) did not maintain optimal interactions with the receptors, leading to reduced binding affinities. On the contrary, a 3‐thienyl group (**11**) proved to be a good alternative for the *N*‐phenyl substituent, increasing binding affinity and affording the most potent compound of the series. Hydrophilic substituents on the aniline nitrogen (**13**–**15**) were not tolerated at the MT_1_ receptor, while a hydroxyl group (**14**, **15**) produced a limited reduction of binding affinity at the MT_2_ receptor. Considering phenyl‐alkylamide derivatives, the phenethyl group (**12**) could be accommodated at the MT_2_ receptor only, while 6‐substituted‐3‐methoxyanilines (**18**, **19**) maintained good binding affinities at both receptors. The potency and MT_2_‐selectivity observed for N‐phenyl‐N‐methyl‐ethylamides **18** and **19** can be explained on the basis of their structural similarity with the lead UCM765. Superposition of docking solutions obtained for UCM765 and compound **18** in the MT_2_ receptor crystal structure highlights the same binding site occupation and interactions (Figure 2, left).

The selectivity for the MT_2_ receptor shown by compounds in Table 1 is consistent with the presence of bulky substituents on the aniline nitrogen atom,^[22]^ or on the neighboring carbon, and with the three‐dimensional structure of the MT_1_ and MT_2_ binding sites. The two receptors are highly similar, with fully conserved ligand‐interacting residues.^[17]^ However, the MT_2_ receptor has a bigger cavity accommodating the substituent in position 2 of melatonin in crystal structures, and that is occupied by the bulky substituent in our docking studies (Figure 2, left). Despite being a lipophilic region, hydroxyl substituents are tolerated and lead to a limited reduction in potency at the MT_2_ receptor. This tolerance for hydrophilic substituents was observed also for other melatonergic ligands, such as 3‐[(3‐hydroxymethyl)phenyl]agomelatine,^[33]^ or 2‐[(3‐hydroxymethyl)phenyl]benzofuran derivatives^[34]^ that can accommodate the hydroxyl group in the same region of the binding site. Compound **14** was docked into the MT_1_ and MT_2_ receptors and the stability of the binding poses was evaluated during a 500 ns‐long molecular dynamics (MD) simulation. The MT_1_ and MT_2_ docking poses were highly similar, with the methoxy group and the amide side chain interacting with Asn4.60 and the Gln residue in extracellular loop 2 (ECL2), respectively, as observed for melatonin in the co‐crystallized complexes. However, MD simulations highlighted a different stability of ligand interactions in the two complexes. In the MT_2_ receptor, the hydrogen bond of the methoxy group with Asn175^4.60^ was rapidly lost, allowing the compound to move toward transmembrane helix (TM) 2 and TM3 and the hydroxyl group to stably interact with Tyr94^2.58^ (Figure 2, right). The hydrogen bond between the amide carbonyl and Gln194 in ECL2 was maintained. On the contrary, the complex between compound **14** and the MT_1_ receptor was quite unstable. Hydrogen bonds with Asn161^4.60^ and Gln181 (ECL2) were frequently lost during MD simulation, with no significant additional interactions with Tyr81^2.58^ (Supporting Information Figure S1). The hydrogen bond undertaken by the hydroxyl group with the MT_2_ receptor, combined with the loss of methoxy group interactions, can justify the limited reduction of MT_2_ binding affinity and the selectivity for this receptor subtype observed for compound **14**.

### Physicochemical characterization

Solubility, metabolic stability and lipophilicity of the new compounds were experimentally evaluated and are reported in Table 2. Thermodynamic solubility was measured at physiological pH and in acidic conditions (pH=1.0). In general, diarylamino derivatives had similar solubility at the two pH values, usually slightly higher at pH=1.0. The most soluble compounds were the primary urea **6 a**, the hydrophilic and basic 4‐pyridyl derivative **13** and the *m*‐hydroxymethyl‐substituted compound **14**. Compound **14** had a solubility greater than 1 mg/mL at both pH values. A higher solubility at acidic than at neutral pH was observed for phenylalkylamides **12**, **18** and **19**, with a solubility >1 mg/mL at pH=1.0. Compared to UCM765, compound **19** was significantly more soluble also at pH=7.4. Metabolic stability was evaluated in the presence of rat and human liver microsomes and expressed as pseudo half‐life and as intrinsic clearance in Table 2. All the compounds were susceptible, albeit at different degrees, to oxidative metabolism, with the exception of the 4‐pyridyl derivative **13** which remained unaltered after 60 min of incubation with microsomes of both species. The primary urea **6 a** was significantly more stable than UCM765 and displayed half‐lives close to those of the metabolically protected UCM924.^[23]^ Alkyl substitution of the urea group (**6 b** and **6 c**) contributed to reduce the metabolic stability in microsomes of both species, which appeared to be inversely related to compound lipophilicity (Log*D*_oct,7.4_). The hydroxy‐substituted derivatives **14** and **15** showed a slight increase (approximately 1.5 fold) of both rat and human microsomal stability, compared to the unsubstituted precursor UCM765. Besides the primary urea **6 a**, the most stable derivatives were compounds **10** and **19**. Compound **10** is a structural analogue of the metabolically protected UCM924, having similar half‐lives. Compound **19** is significantly less lipophilic that UCM765 and lacks the metabolically labile phenyl ring, contributing to its improved microsomal stability.

**Table 2:** Solubility, rat and human microsomal stability and lipophilicity of melatonergic ligands.

Compd.	Solubility pH 1.0 [μg/mL]	Solubility pH 7.4 [μg/mL]	*t*_1/2_ [min] Rat LM^[a]^	CLi^[b]^ Rat LM^[a]^	*t*_1/2_ [min] Human LM^[a]^	CLi^[b]^ Human LM^[a]^	Log *D*_oct,7.4_
**2** (UCM765)	125±5	104±5	1.7±0.4	408±85	40.8±1.1	17±1	2.93±0.01
**3** (UCM924)	6.9±0.1	5.1±0.2	18.2±2.8	38.1±6.0	71.2±3.4	9.7±0.5	3.84±0.05
**6 a**	363.9±3.7	311.4±2.6	15.7±0.4	44.2±1.0	77.5±13.4	9.1±1.5	2.65±0.02
**6 b**	14.9±0.6	10.2±1.7	7.1±0.5	98.1±6.8	15.4±1.0	45.1±3.0	3.30±0.02
**6 c**	17.7±0.9	14.3±0.1	3.2±0.3	218.4±21.2	7.4±1.2	95.7±14.7	3.91±0.02
**10**	54.9±1.1	40.6±0.1	12.2±1.5	58.0±8.2	55.4±6.8	12.8±1.3	3.45±0.02
**11**	161.5±4.4	92.5±3.5	4.0±0.5	175.4±22.3	40.1±5.3	17.5±2.4	2.74±0.01
**12**	>1000^[d]^	109.6±6.9	2.9±0.5	247.3±34.2	7.2±0.5	96.7±6.3	3.44±0.02
**13**	>1000^[d]^	1019.6±93.7	100.9 % ±3.5^[c]^	N.D.	101.5 % ±2.7^[c]^	N.D.	0.41±0.01
**14**	>1000^[d]^	>1000^[d]^	2.9±0.3	241.4±28.5	64.9±6.9	10.8±1.2	2.04±0.01
**15**	95.9±4.2	118.7±2.8	3.3±0.6	217.3±44.6	66.2±2.8	10.5±0.4	2.42±0.02
**18**	>1000^[d]^	339.5±9.7	1.5±0.1	468.9±12.9	11.2±0.7	61.9±3.8	3.11±0.02
**19**	>1000^[d]^	822.9±34.7	17.6±1.5	39.6±3.1	78.5±12.2	8.9±1.4	1.85±0.01

[a] LM: Liver Microsomes. [b] CLi=Intrinsic Clearance (μL min^−1^ mg prot^−1^). [c] Percentage of compound left after 60 min, 37 °C. [d] Weighted compound completely dissolved in the chosen buffer at 1000 μg/mL. N.D.: not determined. Experiments were performed in triplicate and values are reported as the mean±standard deviation.

Taking into consideration binding affinity, solubility and metabolic stability, compounds **14** and **19** were selected for further characterization. The *m*‐hydroxymethyl derivative **14** maintained an MT_2_‐selective profile, with high receptor binding affinity. Its limited metabolic stability could be improved preparing suitable prodrugs, temporarily masking the hydroxyl group, likely responsible for the short half‐life. Additionally, compound **14** is highly soluble at both acidic and neutral pH values. The *p‐*bromo‐phenylalkylamide **19** is a potent ligand, endowed with high solubility and increased metabolic stability. MT_1_ and MT_2_ melatonin receptors, as well as many other GPCRs are capable of downstream signaling through distinct noncanonical pathways such as β‐arrestin in addition to the canonical G protein‐dependent pathways. Therefore the functionality of melatonin ligands is largely depended on the pathway studied.[^35^, ^36^]

The two novel melatonin receptor ligands **14** and **19** have been only partially characterized in terms of functionality and the results are shown in Table 3. Compound **19** showed a full agonist profile, with high potency at the MT_2_ receptor, close to that of melatonin (0.17 nM). In the cAMP functional assay compound **14** behaved as an MT_2_‐selective partial agonist, recalling the behavior observed for UCM765.^[22]^

**Table 3:** Intrinsic activity of select *N*‐(anilinoethyl)amides.

Compd.	MT_1_ EC_50_ [nM]^*[a]*^	*E*_max_ [%]^[b]^	MT_2_ EC_50_ [nM]^[c]^	*E*_max_ [%]^[b]^
**14**	53±0.5	32±3	6.8±0.3	71±2
**19**	4.4±0.2	94±4	0.83±0.05	93±2

[a] EC_50_ values obtained from a cell impedance assay. [b] The Emax values are referred to the % of melatonin response. [c] EC_50_ values obtained from a cAMP assay. Experiments were performed in triplicate and values are reported as the mean±standard deviation.

### Pharmacokinetic evaluation

**Figure 3:**
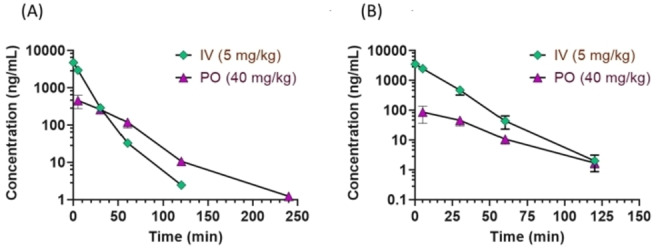
Plasma concentration (semilogarithmic scale) profiles of compounds **19** (A) and **14** (B) in Sprague‐Dawley rats after single intravenous (5 mg kg^−1^) or oral (40 mg kg^−1^) administration.

To evaluate the in vivo behavior of the two melatonergic ligands **14** and **19**, a preliminary pharmacokinetic (PK) study was carried out in male Sprague‐Dawley rats. The compounds were administered as intravenous bolus (IV) or by oral gavage (PO) at doses of 5 or 40 mg kg^−1^, respectively. Quantitative analysis was carried out by UPLC/MS/MS and the plasma concentration profiles are shown in Figure 3, while the main PK parameters are listed in Table 4.

**Table 4:** Main pharmacokinetic parameters in rat plasma obtained for compounds **14** and **19**.

	IV bolus (5 mg kg^−1^)		PO gavage (40 mg kg^−1^)	
Parameter^[a]^	**14**	**19**	**14**	**19**
AUC _IV o‐∞_ [ng min mL^−1^]	66508.2 ±9656.1	66017.0 ±7842.4	2626.5 ±1037.9	19129.8 ±4802.3
C_max_ [ng mL^−1^]	2757.5 ±478.6	2973.0 ±383.9	75.6 ±39.7	455.7 ±178.5
*t*_1/2_ [min]	11.5 ±1.0	13.5 ±0.3	19.8 ±1.9	26.2 ±0.9
CL [mL min^−1^kg^−1^]	76.2 ±10.3	76.4 ±8.8	–	–
*V*_ss_ [L kg^−1^]	0.9 ±0.2	0.7 ±0.1	–	–
*F* [%]	–	–	0.5	3.6

[a] AUC: area under the plasma concentration‐time curve of the drug; *t*_1/2_: half‐life; CL: volume of plasma cleared of the drug per unit time; V_ss_: volume of distribution at the steady state defined by the amount of drug in the body over the concentration of the drug in the plasma at the steady state; *F*: percentage of the dose reaching blood circulation after oral administration.

Upon single IV administration they displayed limited plasma half‐life (compound **14**, 11.5 min; compound **19**, 13.5 min), and a moderately‐high plasma clearance (**14**, 76.2 mL min^−1^kg^−1^; **19**, 76.4 mL min^−1^kg^−1^). For **14** the Cmax was 2757.5 ng mL^−1^ while for **19** it was 2973.0 ng mL^−1^. The steady‐state volume of distribution (V_ss_) was moderate (**14**, 0.9 L kg^−1^; **19**, 0.7 L kg^−1^) indicating a moderate propensity to distribute out from the plasma compartment. After PO administration their plasma half‐life was longer (**14**, 19.8 min; **19**, 26.2 min). with corresponding Cmax of 75.6 mg mL^−1^ (**14**), and 455.7 ng mL^−1^ (**19**). The oral bioavailability was very low for **14** and moderate for **19**. The PK profile of compound **19**, albeit far from optimal, was more favorable than that of compound **14**, with greater oral absorption and longer plasma exposure, likely related to its higher in vitro liver microsomal stability.

## Conclusion

N‐(anilinoethyl)amide melatonergic ligands were designed and synthesized to improve physicochemical properties and metabolic stability of the MT_2_‐selective partial agonist UCM765 which showed sleep promoting effects in rats, but has limited solubility and high propensity to metabolic transformation. Different strategies were applied, including replacement of metabolically liable substituents, insertion of hydrophilic groups and scaffold modification. In vitro studies evidenced compounds with good receptor binding affinities, such as the thienyl derivative **11**, the *meta*‐hydroxymethyl analog **14** or the *p*‐Br‐phenylalkylamide derivative **19**, with some of them endowed with high water solubility and improved microsomal stability. Combined application of analytical techniques, structure‐activity relationship analysis, chemical synthesis and binding studies enabled the selection of compounds **14** and **19** for a preliminary PK study. The *p*‐Br‐phenylalkylamide derivative **19** possesses the most favorable PK profile, showing greater plasma exposure, even if its half‐life remains short. Compound **19** can be considered worthy of consideration for further pharmacological investigation and chemical optimization.

## Experimental Section

**General procedures**. Melting points were determined on a Buchi B‐540 capillary melting point apparatus and are uncorrected. ^1^H NMR and ^13^C NMR spectra were recorded on a Bruker AVANCE 200 or 400, using CDCl_3_ as solvent unless stated otherwise. Chemical shifts (δ scale) are reported in parts per million (ppm) relative to the central peak of the solvent; coupling constants (*J*) are given in hertz (Hz). ESI MS spectra were taken on a Waters Micromass ZQ instrument; molecular ions [M+H]^+^ are given. High‐resolution mass spectroscopy was performed on a Micromass Q‐ToF Micro mass spectrometer (Micromass, Manchester, UK) using an ESI source. The purity of tested compounds, determined by high performance liquid chromatography (HPLC), was greater than 95 % (Supporting Information, Figure S14). These analyses were performed on a Waters HPLC/DAD/MS system (separation module Alliance HT2795, Photo Diode Array Detector 2996, mass detector Micromass ZQ; software: MassLynx 4.1). Column chromatography purifications were performed under “flash” conditions using Merck 230–400 mesh silica gel. Analytical thin‐layer chromatography (TLC) was carried out on Merck silica gel 60 F_254_ plates.

3‐Methoxy‐*N*‐phenylaniline (**4 a**), 3‐methoxyaniline (**4 b**), 1‐(benzyloxy)‐3‐bromobenzene, 3‐(difluoromethyl)bromobenzene, 3‐bromothiophene, 2‐bromo‐5‐methoxyaniline (**4 i**) and 4‐bromopyridine^.^HCl were purchased from commercial suppliers and used without further purification.

**1‐{2‐[(3‐Methoxyphenyl)phenylamino]ethyl}urea (6 a)**. A solution of the nitrile **5** ^[22]^ (262 mg, 1.1 mmol) in THF (7 mL) and 1 M NH_3_ in EtOH (1.5 mL) was hydrogenated over Raney nickel at 4 atm of H_2_ for 6 h at 60 °C. The catalyst was filtered on a celite pad, the filtrate was concentrated *in vacuo*, to give a crude oily amine which was used in the next step without any further purification. A solution of KOCN (150 mg, 1.85 mmol) in H_2_O (0.85 mL) was added dropwise to a solution of the above crude amine in THF‐H_2_O‐AcOH (1–1–0.5 mL) and the resulting mixture was stirred at room temperature for 4 h. The reaction mixture was neutralized with a saturated aqueous solution of NaHCO_3_, and then extracted with EtOAc. The combined organic phases were washed with brine, dried (Na_2_SO_4_) and concentrated under reduced pressure to give a crude residue that was purified by flash chromatography (silica gel; EtOAc as eluent). White solid, mp 78–9 °C (diethyl ether‐petroleum ether), 45 % yield. ^1^H NMR (400 MHz, CDCl_3_) δ: 3.34 (bt, 2H, *J*=6.5 Hz), 3.73 (s, 3H), 3.80 (t, 2H, *J*=6.5 Hz), 4.57 (brs, 2H), 5.39 (brs, 1H), 6.47 (dd, 1H, *J*=2.5 and 8.0 Hz), 6.54–6.57 (m, 2H), 6.96–7.00 (m, 1H), 7.03–7.06 (m, 2H), 7.13 (dd, 1H, *J_1_* =*J_2_* =8.0 Hz), 7.24–7.28 (m, 2H). ^13^C NMR (100 MHz, CDCl_3_) δ: 38.2, 51.8, 55.2, 106.1, 106.3, 112.7, 122.2, 122.4, 129.5, 130.0, 147.6, 149.3, 159.2, 160.6. ESI MS (*m/z*): 286 [M+H]^+^. HRMS (ESI): *m/z* calculated for C_16_H_20_N_3_O_2_, [M+H]^+^ 286.1556. Found: 286.1555.

**1‐Ethyl‐3‐{2‐[(3‐methoxyphenyl)phenylamino]ethyl}urea (6 b)**. A solution of the nitrile **5** ^[22]^ (262 mg, 1.1 mmol) in THF (7 mL) and 1 M NH_3_ in EtOH (1.5 mL) was hydrogenated over Raney nickel at 4 atm of H_2_ for 6 h at 60 °C. The catalyst was filtered on a celite pad, the filtrate was concentrated *in vacuo*, to give the crude oily *N* ^*1*^‐3‐(methoxyphenyl)‐*N* ^*1*^‐phenylethane‐1,2‐diamine which was used in the next step without any further purification. Ethyl isocyanate (1.2 mmol) was added to a solution of the above crude amine in DCM (4 mL) and the resulting mixture was stirred at room temperature for 30 min. The solvent was removed by distillation under reduced pressure to give a crude residue that was purified by flash chromatography (silica gel, EtOAc/cyclohexane 1 : 1 as eluent). White solid, mp 96–7 °C (diethyl ether); 87 % yield. ^1^H NMR (400 MHz, CDCl_3_) δ: 1.06 (t, 3H, *J*=7.0 Hz), 3.11 (q, 2H, *J*=7.0 Hz), 3.38 (t, 2H, *J*=6.5 Hz), 3.73 (s, 3H), 3.82 (t, 2H, *J*=6.5 Hz), 4.69 (brs, 1H), 4.90 (brs, 1H), 6.48 (dd, 1H, *J*=2.5 and 8.0 Hz), 6.53–6.55 (m, 1H), 6.59 (dd, 1H, *J*=2.5 and 8.0 Hz), 6.96–7.00 (m, 1H), 7.05–7.07 (m, 2H), 7.15 (dd, 1H, *J_1_* =*J_2_* =8.0 Hz), 7.24–7.28 (m, 2H). ^13^C NMR (100 MHz, CDCl_3_) δ: 15.4, 35.3, 38.3, 52.0, 55.2, 106.1, 106.2, 112.7, 122.0, 122.2, 129.5, 130.0, 147.6, 149.3, 158.4, 160.6. ESI MS (*m/z*): 314 [M+H]^+^. HRMS (ESI): *m/z* calculated for C_18_H_24_N_3_O_2_, [M+H]^+^ 314.1869. Found: 314.1853.

**1‐{2‐[(3‐Methoxyphenyl)phenylamino]ethyl}‐3‐propylurea (6 c)**: prepared following the above described procedure for the synthesis of **6 b**, using *n*‐propyl isocyanate instead of ethyl isocyanate. White solid, mp 63–5 °C (diethyl ether‐petroleum ether); 91 % yield. ^1^H NMR (400 MHz, CDCl_3_) δ: 0.79 (t, 3H, *J*=7.5 Hz), 1.32–1.41 (m, 2H), 2.95 (t, 2H, *J*=7.0 Hz), 3.31 (t, 2H, *J*=6.5 Hz), 3.65 (s, 3H), 3.75 (t, 2H, *J*=6.5 Hz), 4.60 (brs, 1H), 4.71 (brs, 1H), 6.40 (dd, 1H, *J*=2.5 and 8.0 Hz), 6.45–6.46 (m, 1H), 6.51 (dd, 1H, *J*=2.5 and 8.0 Hz), 6.88–6.90 (m, 1H), 6.92–6.99 (m, 2H), 7.06 (dd, 1H, *J_1_* =*J_2_* =8.0 Hz), 7.16–7.20 (m, 2H). ^13^C NMR (100 MHz, CDCl_3_) δ: 11.3, 23.3, 38.4, 42.3, 52.1, 55.2, 106.2, 106.3, 112.7, 122.0, 122.2, 129.4, 130.0, 147.6, 149.3, 158.5, 160.6. ESI MS (*m/z*): 328 [M+H]^+^. HRMS (ESI): *m/z* calculated for C_19_H_26_N_3_O_2_, [M+H]^+^ 328.2025. Found: 328.2013.

**3‐Benzyloxy‐*N*‐(3‐methoxyphenyl)aniline (4 d)** A Schlenk flask was charged with 3‐methoxyaniline **4 b** (123 mg, 1 mmol) and 1‐(benzyloxy)‐3‐bromobenzene (264 mg, 1 mmol). Dry toluene (10 mL) was added, followed by *t‐*BuONa (144 mg, 1.5 mmol), Pd_2_(dba)_3_ (18 mg, 0.02 mmol) and XantPhos (34 mg, 0.06 mmol). The mixture was evacuated and purged with argon (3 cycles), then heated to 100 °C under argon for 18 h. The mixture was cooled to room temperature, quenched by addition of water and then extracted with EtOAc. After drying over Na_2_SO_4_, the combined organic layers were concentrated under reduced pressure and the resulting crude product was purified by flash chromatography (silica gel, cyclohexane/EtOAc 8 : 2 as eluent). Amorphous solid, 88 % yield. ^1^H NMR (200 MHz, CDCl_3_) δ: 3.78 (s, 3H), 5.05 (s, 2H), 5.73 (brs, 1H), 6.49–6.74 (m, 5H), 7.13–7.43 (m, 8H). ESI MS (*m/z*): 306 [M+H]^+^.

**3‐(Difluoromethyl)‐*N*‐(4‐fluorophenyl)aniline (4 e)**. A Schlenk flask was charged with Pd(OAc)_2_ (14 mg, 0.06 mmol), (±)‐BINAP (41 mg, 0.06 mmol), *t‐*BuOK (190 mg, 1.7 mmol) 3‐(difluoromethyl)bromobenzene (250 mg, 1.2 mmol) and 4‐fluoroaniline (133 mg, 1.2 mmol) under nitrogen atmosphere. Dry toluene (2 mL) was added via syringe and after the addition was completed the mixture was stirred at 100 °C for 3 h. After cooling to room temperature the reaction mixture was quenched with water and then extracted with DCM. The combined organic phases were dried over Na_2_SO_4_ and evaporated under reduced pressure to yield a crude product which was purified by flash chromatography (silica gel, cyclohexane/EtOAc 9 : 1 as eluent). Oil, 44 % yield. ^1^H NMR (200 MHz, CDCl_3_) δ: 5.70 (brs, 1H), 6.58 (t, 1H, *J*=56.5 Hz), 7.03–7.14 (m, 7H), 7.28–7.36 (m, 1H). ESI MS (*m/z*): 238 [M+H]^+^.

***N*** **‐(3‐Methoxyphenyl)thiophen‐3‐amine (4 f)**. An oven dried Schlenk tube containing a magnetic stirrer bar was evacuated and backfilled with argon. The tube was then charged with Pd_2_(dba)_3_ (11 mg, 0.012 mmol), 2‐dicyclohexylphosphino‐2′,6′‐diisopropoxybiphenyl (RuPhos, 23 mg, 0.05 mmol), and *t*‐BuONa (82 mg, 0.85 mmol). The tube was evacuated and argon backfilled once and capped with a septa. 3‐Bromothiophene (99 mg, 58 μl, 0.61 mmol) and 3‐methoxyaniline **4 b** (91 mg, 83 μl, 0.74 mmol) were added via syringe followed by toluene (1.25 mL). The tube was sealed under a positive pressure of argon with a Teflon screw cap and placed into a pre‐hated oil bath (100 °C) for 18 h. The reaction mixture was cooled to room temperature and filtered through a celite pad washing with EtOAc. The solvent was evaporated under reduced pressure and the residue was purified by flash chromatography (silica gel, cyclohexane/EtOAc 9 : 1 as eluent). Oil, 92 % yield. ^1^H NMR (200 MHz, CDCl_3_) δ: 3.79 (s, 3H), 5.71 (brs, 1H), 6.44 (dd, 1H, *J*=2.0 and 8.5 Hz), 6.55–6.58 (m, 2H), 6.77–6.79 (m 1H), 6.94 (dd, 1H, *J*=1.5 and 5.0 Hz), 7.16 (dd, 1H, *J_1_* =*J_2_* =8.5 Hz), 7.25–7.29 (m,1H). ESI MS (*m/z*): 206 [M+H]^+^.

***N*** **‐(3‐Methoxyphenyl)‐2‐phenylacetamide**. A solution of 2‐phenylacetyl chloride (0.4 mL, 2.47 mmol) in DCM (3.5 mL) was added dropwise to an ice‐cooled solution of 3‐methoxyaniline **4 b** (300 mg, 2.44 mmol) and TEA (0.51 mL, 3.66 mmol) in DCM (3.5 mL), and the resulting reaction mixture was stirred at room temperature for 30 min. After dilution with DCM the mixture was washed with a saturated aqueous solution of NaHCO_3_ followed by brine. After drying over Na_2_SO_4_, the solvent was removed by distillation to give a crude residue that was purified by flash chromatography (silica gel; cyclohexane/EtOAc 8 : 2 as eluent). White solid, 84 % yield. Physicochemical data are in agreement with those previously reported.^[37]^

**3‐Methoxy‐*N*‐phenethylaniline (4 g)**. Solid LiAlH_4_ (154 mg, 3.95 mmol) was added portionwise to a stirred ice‐cooled solution of *N*‐(3‐methoxyphenyl)‐2‐phenylacetamide (320 mg, 1.33 mmol) in dry THF (15 mL) under nitrogen atmosphere. Upon completion of the addition, the mixture was refluxed for 1 h. The unreacted LiAlH_4_ was destroyed by careful addition of water at 0 °C, and the resulting mixture was filtered through a celite pad. The filtrate was concentrated *in vacuo*, and the residue was partitioned between EtOAc and water. The combined organic phases were washed with brine, dried (Na_2_SO_4_), and concentrated by distillation under reduced pressure to give a crude residue which was purified by flash chromatography (silica gel, cyclohexane/EtOAc 8 : 2 as eluent). Oil; 70 % yield. Physicochemical data are in agreement with those previously reported.^[38]^

**4‐Methoxy‐[1,1’‐biphenyl]‐2‐amine (4 h)**. A solution of 4‐methoxy‐2‐nitro‐1,1’‐biphenyl^[27]^ (530 mg. 2.31 mmol) in a 1 : 1 mixture of EtOAc and EtOH (46 mL) was hydrogenated over 10 % Pd−C (43 mg) at 4 atm of H_2_ for 6 h at room temperature. The catalyst was filtered on a celite pad, the filtrate was concentrated *in vacuo*, to give the corresponding amine, which was used without any further purification. Oil, 99 %. Physicochemical data are in agreement with those previously reported.^[39]^

**General procedure for reductive N‐alkylation of anilines**. TFA (1 mL) and TES (0.4 mL, 2.5 mmol) were added to a solution of the opportune aniline (1 mmol) and *N‐*(2,2‐dimethoxyethyl)acetamide^[40]^ (206 mg, 1.4 mmol) in DCM (2 mL), and the resulting mixture was stirred at room temperature for 2 h under a nitrogen atmosphere. After cooling to 0 °C, the reaction mixture was carefully neutralized with NaHCO_3_ aqueous saturated solution of and diluted with DCM. The aqueous phase was extract with DCM and the combined organic phases were washed with brine, and dried over Na_2_SO_4_. The solvent was removed by distillation, and the crude residue was purified by column chromatography to afford the desired compound.

**Methyl 3‐[(2‐acetamidoethyl)(3‐methoxyphenyl)amino) benzoate (8)** was prepared following the above‐described general procedure starting from **4 c**.^[41]^ Flash chromatography: silica gel, EtOAc as eluent. Oil, 81 % yield. ^1^H NMR (200 MHz, CDCl_3_) δ: 3.80 (s, 3H), 3.91 (s, 3H), 5.83 (brs, 1H), 6.54 (dd, 1H, *J*=2.0 and 8.0 Hz), 6.65–6.71 (m, 2H), 7.21 (dd, 1H, *J_1_* =*J_2_* =8.0 Hz), 7.26–7.38 (m, 2H), 7.58–7.61 (m, 1H), 7.74–7.75 (m, 1H). ESI MS (*m/z*): 258 [M+H]^+^.

***N*** **‐{2‐[(3‐Benzyloxyphenyl)(3‐methoxyphenyl)amino]ethyl} acetamide (9)** was prepared following the above‐described general procedure starting from **4 d**. Flash chromatography: silica gel, EtOAc as eluent. Oil, 80 % yield. ^1^H NMR (400 MHz, CDCl_3_) δ: 1.92 (s, 3H), 3.49 (m, 2H), 3.77 (s, 3H), 3.86 (t, 2H, *J*=6.5 Hz), 5.02 (s, 2H), 5.62 (brs, 1H), 6.60 (dd, 1H, *J*=2.5 and 8.0 Hz), 6.60–6.63 (m, 2H), 6.65–6.66 (m, 2H), 7.17–7.21 (m, 2H), 7.32–7.41 (m, 6H). ESI MS (*m/z*): 391 [M+H]^+^.

***N*** **‐(2‐{[3‐(Difluoromethyl)phenyl](4‐fluorophenyl)amino}ethyl)acetamide (10)** was prepared following the above‐described general procedure starting from **4 e**. Flash chromatography: silica gel, EtOAc/cyclohexane 7 : 3 as eluent. White solid, mp 76–7 °C (diethyl ether‐petroleum ether); 77 % yield. ^1^H NMR (400 MHz, CDCl_3_) δ: 1.95 (s, 3H), 3.48 (m, 2H), 3.85 (t, 2H, *J*=6.5 Hz), 5.80 (brs, 1H), 6.54 (t, 1H, *J*=56.5 Hz), 6.93–6.97 (m, 3H), 7.04–7.14 (m, 4H), 7.25–7.60 (m, 1H). ^13^C NMR (100 MHz, CDCl_3_) δ: 23.1, 37.6, 51.3, 113.4 (t, *J*=6.2 Hz), 114.8 (t, *J*=237.2 Hz), 116.4 (t, *J*=6.2 Hz), 116.7 (d, *J*=22.3 Hz), 118.8 (t, *J*=1.8 Hz), 126.7 (d, *J*=8.2 Hz), 129.7, 135.5 (t, *J*=21.8 Hz), 142.8 (d, *J*=3.0 Hz), 148.7, 159 7 (d, *J*=43.0 Hz), 170.6. ESI MS (*m/z*): 323 [M+H]^+^. HRMS (ESI): *m/z* calculated for C_17_H_18_F_3_N_2_O, [M+H]^+^ 323.1371. Found: 323.1359.

***N*** **‐{2‐[(3‐Methoxyphenyl)(thiophen‐3‐yl)amino]ethyl}acetamide (11)** was prepared following the above‐described general procedure starting from **4 f**. Flash chromatography: silica gel, EtOAc as eluent. Yellowish solid, mp 76–7 °C (diethyl ether‐petroleum ether); 66 % yield. ^1^H NMR (400 MHz, CDCl_3_) δ: 1.93 (s, 3H), 3.50 (m, 2H), 3.77 (s, 3H), 3.83 (t, 2H, *J*=6.5 Hz), 5.71 (brs, 1H), 6.47 (dd, 1H, *J*=2.5 and 8.0 Hz), 6.54–6.56 (m, 1H), 6.59 (dd, 1H, *J*=2.5 and 8.0 Hz), 6.70–6.78 (m, 1H), 6.88 (d, 1H. *J*=5.0 Hz), 7.15 (t, 1H, *J_1_* =*J_2_=*8.0 Hz), 7.25 (d, 1H, *J*=5.0 Hz). ^13^C NMR (100 MHz, CDCl_3_) δ: 23.2, 37.8, 52.0, 55.2, 104.7, 105.8, 110.4, 111.0, 124.1, 125.4, 129.9, 146.4, 149.5, 160.6, 170.4. ESI MS (*m/z*): 291 [M+H]^+^. HRMS (ESI): *m/z* calculated for C_15_H_19_N_2_O_2_S, [M+H]^+^ 291.1167. Found: 291.1167.

***N*** **‐{2‐[(3‐Methoxyphenyl)(phenethyl)amino]ethyl}acetamide (12)** was prepared following the above‐described general procedure starting from **4 g**. Flash chromatography: silica gel, EtOAc as eluent. Oil, 36 % yield. ^1^H NMR (400 MHz, CDCl_3_) δ: 1.83 (s, 3H), 2.79 (t, 3H, *J*=7.5 Hz), 3.23–3.27 (m, 4H), 3.45 (t, 2H, *J*=7.5 Hz), 3.74 (s, 3H), 5.40 (brs. 1H), 6.21–6.38 (m, 3H), 7.06–7.25 (m, 6H). ^13^C NMR (100 MHz, CDCl_3_) δ: 23.2, 33.3, 37.5, 50.8, 53.1, 55.2, 99.5, 101.8, 105.9, 126.4, 128.6, 128.9, 130.2, 139.5, 149.2, 161.0, 170.3. ESI MS (*m/z*): 313 [M+H]^+^. HRMS (ESI): *m/z* calculated for C_19_H_24_N_2_O_2_Na, [M+H]^+^ 335.1735. Found: 335.1747.

***N*** **‐{2‐[(3‐Methoxyphenyl)(pyridine‐4‐yl)amino]ethyl}acetamide (13)**: A Schlenk flask was charged with 4‐bromopyridine^.^HCl (100 mg, 0.48 mmol) and *N*‐{2‐[(3‐methoxyphenyl)amino]ethyl}acetamide **7** (121 mg, 0.62 mmol).^[22]^ Dry toluene (5 mL) was added, followed by *t*‐BuONa (115 mg, 1.2 mmol), Pd_2_(dba)_3_ (9 mg, 0.01 mmol) and XantPhos (17 mg, 0.03 mmol). The mixture was evacuated and purged with argon (3 cycles), then heated to 100 °C for 16 h under argon. The mixture was cooled to room temperature, quenched by addition of water and then extracted with EtOAc. After drying over Na_2_SO_4_, the combined organic layers were concentrated by distillation under reduced pressure and the resulting crude product was purified by filtration on a pad of silica gel (DCM/MeOH 9 : 1 as eluent). Oil; 95 % yield. ^1^H NMR (400 MHz, CDCl_3_) δ: 1.90 (s, 3H), 3.45 (m, 2H), 3.78 (s, 3H), 3.84 (t, 2H, *J*=6.5 Hz), 6.61 (dd, 2H, *J*=1.5 and 5.0 Hz), 6.71 (brt, 1H), 6.73–6.75 (m, 1H), 6.78 (ddd, 1H, *J*=1.0, 2.5 and 8.0 Hz), 6.84 (ddd, 1H, *J*=1.0, 2.5 and 8.0 Hz), 7.33 (t, 1H, *J_1_* =*J_2_* =8.0 Hz). 8.08 (dd, 2H, *J*=1.5 and 5.0 Hz). ^13^C NMR (100 MHz, CDCl_3_) δ: 23.0, 37.3, 50.4, 55.4, 108.6, 112.5, 113.3, 119.5, 131.0, 145.4, 148.9, 153.9, 161.2, 171.0. ESI MS (*m/z*): 286 [M+H]^+^. HRMS (ESI): *m/z* calculated for C_16_H_20_N_3_O_2_, [M+H]^+^ 286.1556. Found: 286.1549.

***N*** **‐{2‐[(3‐Hydroxymethylphenyl)(3‐methoxyphenyl)amino]ethyl} acetamide (14)**. A solution of **8** (150 mg, 0.44 mmol) in dry THF (3 mL) was added dropwise to a stirred ice‐cooled suspension of LiAlH_4_ (34 mg, 0.88 mmol) in dry THF (3 mL) under a nitrogen atmosphere, and the resulting mixture was stirred at 0 °C for 1 h. The unreacted LiAlH_4_ was destroyed by careful addition of water at 0 °C, and the resulting mixture was filtered through a celite pad. The filtrate was concentrated *in vacuo*, and the residue was partitioned between EtOAc and water. The organic phases were combined and washed with brine, dried (Na_2_SO_4_), and concentrated by distillation under reduced pressure to yield a crude residue, which was purified by flash chromatography (silica gel, EtOAc as eluent). Oil, 84 % yield. ^1^H NMR (400 MHz, CDCl_3_) δ: 1.84 (s, 3H), 3.36 (brs, 1H), 3.40 (m, 2H), 3.72 (s, 3H), 3.80 (t, 1H, *J*=6.5 Hz), 4.57 (s, 2H), 6.35 (brt, 1H), 6.46 (ddd, 1H, *J*=1.0, 2.5 and 8.0 Hz), 6.52–6.54 (m, 1H), 6.56 (ddd, 1H, *J*=1.0, 2.5 and 8.0 Hz), 6.90–6.93 (m, 2H), 7.08–7.09 (m, 1H), 7.12 (t, 1H, *J_1_* =*J_2_* =8.0 Hz), 7.20 (t, 1H, *J_1_* =*J_2_* =8.0 Hz). ^13^C NMR (100 MHz, CDCl_3_) δ: 22.9, 37.7, 50.9, 55.2, 64.8, 106.3, 106.4, 112.8, 120.0, 120.7, 120.9, 129.4, 130.0, 142.7, 147.6, 149.1, 106.6, 171.1. ESI MS (*m/z*): 315 [M+H]^+^. HRMS (ESI): *m/z* calculated for C_18_H_22_N_2_O_3_Na, [M+H]^+^ 337.1528. Found: 337.1541.

***N*** **‐{2‐[(3‐Hydroxyphenyl)(3‐methoxyphenyl)amino]ethyl} acetamide (15)**. A solution of **6** (120 mg, 0.4 mmol), in MeOH (10 mL) was hydrogenated over 10 % Pd−C (20 mg, 0.02 mmol) at 1 atm of H_2_ for 2.5 h at room temperature. The catalyst was filtered on a celite pad, the filtrate was concentrated *in vacuo*, and the crude residue was purified by flash chromatography (silica gel, EtOAc as eluent). White solid, mp 110 °C (diethyl ether); 80 % yield. ^1^H NMR (400 MHz, CDCl_3_) δ: 1.93 (s, 3H), 3.47 (m, 2H), 3.76 (s, 3H), 3.83 (t, 2H, *J*=6.5 Hz), 5.76 (brs, 1H), 6.47 (ddd, 1H, *J*=1.0, 2.5 and 8.0 Hz), 6.52 (ddd, 1H, *J*=1.0, 2.5 and 8.0 Hz), 6.55 (ddd, 1H, *J*=1.0, 2.5 and 8.0 Hz), 6.59 (dd, 1H, *J_1_* =*J_2_=*2.5 Hz), 6.61 (dd, 1H, *J_1_* =*J_2_=*2.5 Hz), 6.65 (ddd, 1H, *J*=1.0, 2.5 and 8.0 Hz), 7.09 (dd, 1H, *J_1_* =*J_2_=*8.0 Hz), 7.18 (dd, 1H, *J_1_* =*J_2_=*8.0 Hz). ^13^C NMR (100 MHz, CDCl_3_) δ: 23.1, 38.0, 51.0, 55.3, 107.3, 107.5, 107.8, 108.7, 112.5, 114.3, 130.1, 130.2, 148.8, 149.0, 157.3, 160.7, 171.0. ESI MS (*m/z*): 301 [M+H]^+^. HRMS (ESI): *m/z* calculated for C_17_H_21_N_2_O_3_, [M+H]^+^ 301.1552. Found: 301.1563.

***N*** **‐{2‐[(4‐Methoxy‐[1,1’‐biphenyl]‐2‐yl)amino]ethyl}acetamide (16)** was prepared following the above‐described reductive *N*‐alkylation general procedure starting from **4 h**. Flash chromatography: silica gel, EtOAc as eluent. Amorphous solid, 65 % yield. ^1^H NMR (200 MHz, CDCl_3_) δ: 1.93 (s.3H), 3.23–3.29 (m, 2H), 3.37–3.46 (m, 2H), 3.84 (s, 3H), 4.20 (brs, 1H), 5.65 (brs, 1H), 6.34–6.36 (m, 1H), 6.36 (dd, 1H, *J*=2.5 and 8.0 Hz), 7.03 (d, 1H, *J*=8.0 Hz), 7.31–7.49 (m, 5H). ESI MS (*m/z*): 285 [M+H]^+^.

***N*** **‐{2‐[(2‐Bromo‐5‐methoxyphenyl)amino]ethyl}acetamide (17)** was prepared following the above‐described reductive *N*‐alkylation general procedure starting from **4 i**. Flash chromatography: silica gel, EtOAc as eluent. Amorphous solid, 70 % yield. ^1^H NMR (200 MHz, CDCl_3_) δ: 2.02 (s, 3H), 3.29–3.35 (m, 2H), 3.48–3.57 (m, 2H), 3.78 (s, 3H), 4.58 (brs, 1H), 5.84 (brs. 1H), 6.18 (dd, 1H, *J*=3.0 and 9.0 Hz), 6.26 (d, 1H, *J*=3.0 Hz), 7.30 (d, 1H, *J*=9.0 Hz). ESI MS (*m/z*): 287 [M+H]^+^.

***N*** **‐{2‐[(4‐Methoxy‐[1,1’‐biphenyl]‐2‐yl)(methylamino)]ethyl}acetamide (18)**. A suspension of **16** (284 mg, 1 mmol), NaHCO_3_ (84 mg, 1 mmol) and methyl iodide (0.4 mL, 6.5 mmol) in dry MeOH (11 mL) was heated at 50 °C for 24 h. After removing the solvent by distillation *in vacuo*, the residue was poured into water, extracted with EtOAc, and the combined organic phases were washed with brine and dried (Na_2_SO_4_). The solvent was removed by distillation under reduced pressure and the residue was purified by flash chromatography (silica gel, DCM/EtOAc 8 : 2 as eluent). Oil, 74 % yield. ^1^H NMR (400 MHz, CDCl_3_) δ: 1.65 (s, 3H), 2.54 (s, 3H), 2.71 (t, 2H, *J*=6.5 Hz), 3.04 (m, 2H), 3.75 (s, 3H), 5.16 (brs, 1H), 6.61 (dd, 1H, *J*=2.5 and 8.5 Hz), 6.70 (d, 1H, *J*=2.5 Hz), 7.09 (d, 1H, *J*=8.5 Hz), 7.20–7.25 (m, 1H), 7.30–7.36 (m, 4H). ^13^C NMR (100 MHz, CDCl_3_) δ: 23.1, 36.9, 40.8, 55.3, 55.5, 107.6, 108.5, 126.6, 128.3, 129.5, 130.1, 132.0, 141.1, 151.9, 159.9, 170.1. ESI MS (*m/z*): 299 [M+H]^+^. HRMS (ESI): *m/z* calculated for C_18_H_23_N_2_O_2_, [M+H]^+^ 299.1760. Found: 299.1751.

***N*** **‐{2‐[(2‐Bromo‐5‐methoxyphenyl)methylamino]ethyl}acetamide (19)**. Sodium cyanoborohydride (100 mg, 1.20 mmol) and a 37 % HCHO aqueous solution (0.5 mL) were added to a solution of **17** (143 mg, 0.5 mmol), in MeOH (5 mL) and AcOH (to pH=5), and the resulting mixture was stirred at room temperature for 1 h. After removing the solvent by distillation *in vacuo*, the residue was taken up in water and extracted with EtOAc. The combined organic layers were dried (Na_2_SO_4_) and concentrated by distillation under reduced pressure to give a crude residue which was purified by flash chromatography (silica gel, cyclohexane/EtOAc 6 : 4 as eluent). White solid, mp 94–6 °C (diethyl ether‐petroleum ether); 78 % yield. ^1^H NMR (400 MHz, CDCl_3_) δ: 1.97 (s, 3H), 2.70 (s, 3H), 3.09 (t, 2H, *J*=6.0 Hz), 3.39 (m, 2H), 3.78 (s, 3H), 6.30 (brs. 1H), 6.56 (dd, 1H, *J*=3.0 and 9.0 Hz), 6.72 (d, 1H, *J*=3.0 Hz), 7.45 (d, 1H, *J*=9.0 Hz). ^13^C NMR (100 MHz, CDCl_3_) δ: 23.3, 36.8, 41.7, 54.2, 55.5, 109.8, 110.6, 111.7, 133.8, 151.3, 160.0, 170.2. ESI MS (*m/z*): 301 [M+H]^+^. HRMS (ESI): *m/z* calculated for C_12_H_18_BrN_2_O_2_, [M+H]^+^ 301.0552. Found: 301.0552.

### Molecular modelling

*Protein preparation*. The crystal structures of the MT_1_ and MT_2_ receptors in complex with 2‐phenylmelatonin (PDB codes 6ME3^[16]^ and 6ME6,^[17]^ respectively) were prepared for molecular modeling studies. Molecules used as buffer stabilizers in crystallization procedures were removed. Residues belonging to the N‐terminus sequence of the MT_2_ receptor and connecting to apocytochrome *b*_562_RIL were removed as well, leaving Pro36 as the first residue. The intracellular loop 3 (ICL3, residues Gln219‐Pro227 and Arg232‐Leu240 in MT_1_ and MT_2_, respectively) and the missing side chains were added with Modeller 9.21.^[42]^ One hundred models were generated for each structure, leaving residues adjacent to the reconstructed loop flexible to allow proper geometries of the construct (Arg218 and Lys228 for the MT_1_ receptor, Arg231 and Cys241 for the MT_2_ receptor), while the rest of the protein was kept frozen. Models were ranked according to the built‐in molecular probability density function (molpdf)^[43]^ and MT_1_ and MT_2_ structures with the lowest molpdf were selected. Mutations in the crystallized proteins were reverted to wild‐type residues with Maestro 11.6.^[44]^ Hydrogen atoms and termini caps were added with the Protein Preparation Wizard tool of the Maestro Suite.^[45]^ ICL3 residues and the side chains of the modified amino acids were submitted to energy minimization with OPLS3e force field^[46]^ implemented in MacroModel 12.0,^[47]^ using the Polak‐Ribière conjugate gradient method^[48]^ to a convergence threshold of 0.05 kJ ⋅ mol^−1^ ⋅ Å^−1^ to relieve steric and electrostatic clashes. The orientation of thiol and hydroxyl groups and the conformation of asparagine, glutamine, and histidine residues were adjusted to optimize the hydrogen bonding network. Basic and acidic amino acids were modelled in their charged state. The final structures were optimized through a first energy minimization in which only optimization of hydrogen atom positions was allowed, followed by a second minimization with heavy atoms positions restrained to an RMSD value of 0.3 Å, as implemented in the Protein Preparation Wizard tool.^[38]^

*Ligand docking*. Compounds UCM765, **14** and **18** were built in Maestro 11.6^[44]^ and minimized in an implicit water model^[49]^ with the OPLS3e force field implemented in MacroModel 12.0 to an energy gradient of 0.01 kJ ⋅ mol^−1^ ⋅ Å^−1^. Docking grids were generated by imposing a cubic bounding box of 10 Å and an enclosing box of 20 Å, centered on 2‐phenylmelatonin of the prepared MT_1_ and MT_2_ receptor complexes. Docking runs were performed with Glide 7.9^[50]^ in standard precision mode. The scoring window cutoff was increased to 1,000 in order to widen the selection of the initial poses for the rough scoring stage. The MAXKEEP and MAXREF parameters, which control the number of poses to retain after the rough scoring stage and the number of poses to refine, were increased by a tenfold with respect to the default values. The number of poses to be submitted to post‐dock minimization was increased to 200. The best GScore solutions, allowing the formation of hydrogen bonds with Asn162^4.60^ and Gln181^ECL2^ in the MT_1_ receptor and with Asn175^4.60^ and Gln194^ECL2^ in the MT_2_ receptor for the methoxy and amide groups of the ligands, were merged into the protein structure, and the resulting complexes were energy‐minimized with MacroModel 12.0 to an energy gradient of 0.01 kJ ⋅ mol^−1^ ⋅ Å^−1^. During energy minimization the ligands and the residue side chains within 5 Å were free to move, while the protein backbone was kept fixed.

*Molecular dynamics (MD) simulations*. The energy minimized complexes of the MT_1_ and MT_2_ receptors bound to compound **14** were embedded with OPM orientation^[51]^ in a pre‐equilibrated POPC bilayer,^[52]^ setting the dimension of the membrane patch in order to keep the solute molecules distant at least 13 Å from their periodic neighbouring images, and solvated with TIP3P water molecules.^[53]^ The systems’ net charge was neutralized by adding 16 Cl^−^ and 10 Cl^−^ ions, respectively.

Simulations were conducted with Desmond 5.4^[54]^ with the OPLS3e force field, following a protocol already applied to melatonergic ligands.^[55]^ Bond lengths involving hydrogen atoms were constrained to their equilibrium values by applying the M‐SHAKE algorithm.^[56]^ Short‐range electrostatics and van der Waals interactions were truncated above 9 Å, whereas long‐range electrostatic interactions were treated using the smooth Particle Mesh Ewald summation.^[57]^ A RESPA integrator^[58]^ was used with a double time‐step regime: 2 fs for bonded and short‐range non‐bonded forces, 6 fs for long‐range electrostatic interactions. Systems were submitted to various equilibration stages for a total of 6.0 ns of simulation (see the detailed equilibration protocol in the Supporting Information), restraining the ligand heavy atoms onto the position obtained from docking calculations. The production phase, in which the ligand was free to move, lasted for 500 ns in NPT ensemble at 1.0 atm by applying a Langevin coupling scheme^[59]^ with a relaxation time of 1.0 ps at 300 K and with a damping coefficient of 2.0 ps^−1^. Receptor structures were restrained through the application of isotropic force constants of 0.1 kcal ⋅ mol^−1^ ⋅ Å^−2^ on the alpha carbons, while the backbone heavy atoms of the capped terminal residues were restrained with a force constant of 1.0 kcal ⋅ mol^−1^ ⋅ Å^−2^.

### Melatonin receptor binding and intrinsic activity evaluation

The in vitro pharmacology experiments were conducted at Cerep (Celle‐Lévescault, France) following radioligand binding and cellular functional assays developed elsewhere.[^60^, ^61^, ^62^]

Binding affinities were determined using 2‐[^125^I]iodomelatonin as the labeled ligand in competition experiments on cloned human MT_1_ and MT_2_ receptors expressed in CHO cells.[^60^, ^61^] 2‐[^125^I]Iodomelatonin (10 pM for MT_1_ and 50 pM for MT_2_) and different concentrations of tested compounds (10^−11^–10^−7^ M) were incubated with the receptor preparation for 60 min at room temperature (MT_1_) or 90 min at 37 °C (MT_2_). Nonspecific binding was assessed in the presence of melatonin 1 μM. After incubation, the % of radioligand binding inhibition was determined via a scintillation counting method. The radioligand binding experiments were performed in triplicate. IC_50_ values were determined by nonlinear regression analysis of the competition curves using Hill equation curve fitting. The p*K*_i_ values were calculated from the IC_50_ values in accordance with the Cheng‐Prusoff equation.^[28]^

To define the functional activity of the new compounds, cellular functional assays were performed on CHO cells stably expressing human MT_1_ or MT_2_ receptors. Agonist activity at MT_1_ receptors was evaluated using a cell impedance assay measured by cellular dielectric spectroscopy,^[62]^ and at MT_2_ receptors with an cAMP assay, measured by a fluorometric method.^[61]^ The EC_50_ values were determined by non‐linear regression analysis of the concentration‐response curves. Experiments were performed in triplicate.

### Metabolic stability assays in rat and human liver microsomes

Stock solutions of melatonin receptor ligands were prepared in DMSO immediately before use; cosolvent concentration in final samples was kept constant at 1 % v/v. Each compound (final concentration: 5 μM) was incubated in the presence of rat or human liver microsomes (final concentration: 1 mg protein/mL) and of a NADPH‐regenerating system (final concentrations: 2 mM NADP^+^, 10 mM glucose‐6‐phosphate, 0.4 U mL^−1^ glucose‐6‐phosphate dehydrogenase, 5 mM MgCl_2_) in a 10 mM Phosphate Buffered Saline (PBS) solution, pH 7.4, at 37 °C. Compound was added after a 10 min pre‐incubation at 37 °C and. at stated time points, depending on compound kinetics, sample aliquots were withdrawn, metabolic reaction was quenched by acetonitrile addition (1 : 2), and after a centrifugation step (9000 g, 10 min, 4 °C) the supernatant was analyzed by HPLC‐UV‐VIS.

Apparent half‐lives (t_1/2_ in min) for the metabolic clearance of compounds were calculated from the pseudo first‐order rate constants (k) obtained by linear regression of the log chromatographic peak area versus time plots and are reported as means of three experiments±standard deviation. Intrinsic clearance (CLi) values of the compounds were calculated using the following equation: CLi (μL×min^−1^×mg protein^−1^)=k×V where V (μL×mg protein^−1^)=incubation volume/mg protein added.

Pooled rat and human liver microsomes (20 mg/mL) were supplied by BD Biosciences (BD Biosciences, Woburn, MA, USA). Acetonitrile and methanol were HPLC grade and were supplied by Sigma‐Aldrich (Sigma‐Aldrich srl, Milan, Italy). Water was freshly bidistilled before use. All other reagents were purchased in the highest purity available.

### Experimental lipophilicity

Distribution coefficients (log *D*_oct, 7.4_) were determined employing the reference shake‐flask method[^63^, ^64^] in the biphasic *n*‐octanol/water partition system, at room temperature (25±1 °C) and at physiological pH 7.4. Chosen buffer was 50 mM zwitterionic MOPS (3‐morpholinopropanesulfonic acid), pH 7.4 to avoid ionic couple partitioning, with ionic strength adjusted to 0.15 M by KCl addition. Compounds, after partitioning overnight, and dilution of both phases with methanol, were dosed in the HPLC‐UV system, as detailed below. Reported log *D*_oct,7.4_ values are the mean of three measurements±standard deviation.

### Solubility measurements

Thermodynamic solubility values were measured in two different buffers (pH 1.0; pH 7.4) by the shake flask method.^[65]^ 1 mg of each compound was placed in 1 mL of appropriate buffer and shaken at room temperature overnight. The resulting suspension was centrifuged (10 min, 10000 g, 20 °C) and the supernatant analyzed by HPLC‐UV. Solubility values were interpolated from calibration curves prepared from concentrated DMSO stock solutions of each compound. Reported solubility values are the mean of three measurements±standard deviation.

### Sample analysis by HPLC‐UV

A Shimadzu High Performance Liquid Chromatography (HPLC) system coupled to UV‐VIS detection (Shimadzu Corp., Kyoto, Japan) was employed to analyze melatonin receptor ligands in metabolic stability and physicochemical characterization assays. It consisted of a LC‐10ADvp solvent delivery module, a 20 μL Rheodyne sample injector (Rheodyne LLC, Rohnert Park, CA, USA) and a SPD‐10Avp UV‐VIS detector. PeakSimple 2.83 software was employed for data acquisition and HPLC peak integration. HPLC columns were a RP‐C18 Supelco Discovery (Supelco, Bellefonte, PA, USA), 5 μm, 150×4.6 mm i.d. for all compounds except **13**, and a Phenomenex Gemini, 5 μm, 150×4.6 mm i.d. for compound **13** (Phenomenex). Elution conditions were optimized for each compound at a flow rate of 1 mL min^−^1 employing mobile phases consisting of water and acetonitrile at different percentages, while UV detection was set at λ = 254 nm. For compound **13**, the mobile phase consisted of a 10 mM ammonium acetate buffer pH 7.0 and methanol and the UV detection was set at λ = 281 nm.

### In vivo pharmacokinetic studies

The in vivo pharmacokinetic experiments were conducted at NiKem Research (Baranzate – Milan – Italy). PK studies were performed using Sprague‐Dawley CD (albino) male rats (7–9 weeks‐old, weight 160–200 grams, Charles River Lab. Italia, Calco). Animals were housed under standard conditions and had free access to water and standard laboratory rodent diet. Care and husbandry of animals were in conformity with the institutional guidelines, in compliance with national and international laws and policies (EEC Council Directive 86/609, OJL 358 m, 1, Dec. 122, 1987; NIH Guide for the care and Use of Laboratory Animals, NIH Publication No. 86–23, 1985). The compounds were dissolved in water containing 3 % DMSO and 20 % Encapsin (and a stechiometric amount of HCl for **14**) at a concentration of 2.5 mg mL^−1^ for the IV (bolus) dose, or in water containing 5 % DMSO and 10 % encapsin (and a stechiometric amount of HCl for **14**) at a concentration of 4 mg mL^−1^ for PO (gavage) dose. Rats were randomly assigned to four treatment groups (*n*=3) and received a single IV bolus dose (5 mg kg^−1^) or a single oral administration (40 mg kg^−1^) through oral gavage of compound **14** or **19**. Serial blood samples (200 mL) were collected from caudal vein at 5, 30, 60, 120, 480 and 1440 min after IV injection, or at 15, 30, 60, 120, 480 and 1440 min after PO administration. Blood samples were collected in heparinized eppendorfs (Heparin Vister 5000 U.I./mL_Marvecs Pharma), gently mixed and placed on ice; then blood was centrifuged (3500×g, at 4 °C for 15 min), the plasma was collected and immediately frozen at −80 °C until submission to UPLC/MS/MS analysis. For the sample preparation, 100 μL of plasma spiked with 5 μL of internal standard (I.S.; **19** for compound **14** and **14** for compound **19**; 2.5 μg mL^−1^;) were added to a SW96 deep well plate (Waters) containing 300 μL of acetonitrile. The plate was shaken for 10 min and then centrifuged at 3000 rpm for 15 min. UPLC/MS/MS analyses were performed on an Acquity UPLC, coupled with a sample organizer and interfaced with a triple quadrupole Premiere XE (Waters, Milford, USA). LC runs (inj. vol. 5 μL) were carried out at 50 °C on Acquity BEH C_18_ columns (1.7 μm, 2.1×50 mm) at a flow rate of 0.45 mL min^−1^. Mobile phases consisted of a phase A (water) and a phase B (0.1 % formic acid in acetonitrile). The column was conditioned with 2 % phase B for 0.2 min, then brought to 100 % phase B within 0.6 min and maintained at these conditions for 1.1 min. Analyses were carried out using a positive electrospray ionization [ESI(+)] interface in multiple reaction monitoring (MRM) mode. Capillary 3.5 Kv; extractor 3 V; source T 140 °C, desolvation T 450 °C. Transitions: for compound **14** Q1/Q3 315.2/86, CV 14, CE16+315.2/238 CV 14, CE19; for I.S. (compound **19**): 301.1/86 CV 14 CE16; LLOQ: 1 ng ml^−1^. Transitions: for compound **19** Q1/Q3: 301.1/86CV 14 CE16; for I.S. (compound **14**) 315.2/238CV 14, CE19; LLOQ: 1 ng mL^−1^. Pharmacokinetic analysis was performed by non‐compartmental analysis (NCA) using the WinNonlin 5.1 software (Pharsight, Mountain View, CA, USA. Absolute oral bioavailability *(F*) was calculated by linear trapezoidal rule using the relationship: *F*=[dose_IV_×AUC_oral o‐∞_/dose_oral_×AUC_IV o‐∞_]×100.

## Conflict of interest

The authors declare no conflict of interest.

## Biographical Information

*Michele Mari is Assistant Professor (RTD‐B) in Medicinal Chemistry at University of Urbino, working in the field of natural products and melatonin receptor ligands. He achieved his Ph.D. in Chemical and Pharmaceutical Sciences in 2014 from the same university working on the strategic indole functionalization for the synthesis of tryptophan containing alkaloids and biologically active tryptamines. He spent 9 months at ETH Zurich as visiting researcher in the group Prof. Jeffrey W. Bode working on the total synthesis of proteins. He spent 5 years as Postdoctoral Scientist at University of Urbino, in August 2019 he became Assistant Professor in Medicinal Chemistry, which is still his current position. In September 2017 he founded GLUOS with some colleagues, an innovative Start up and University Spin off on the synthesis of molecules for medical devices and he is actually the President of the Board of Directors of the company. He is also member of the organizing committee of the European School of Medicinal Chemistry, Member of the communication teams of the EFMC and Medicinal Chemistry Division of the Italian Chemical Society, Member of the Board of the Young Scientists Network of the EFMC*.

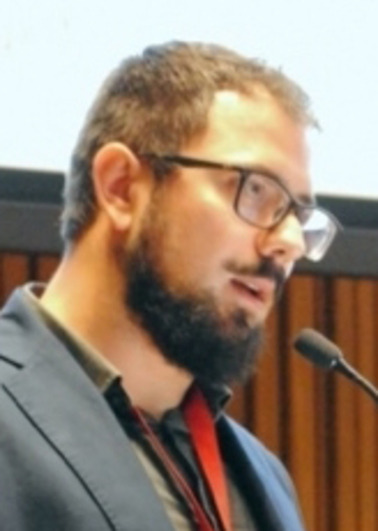

## Supporting information

As a service to our authors and readers, this journal provides supporting information supplied by the authors. Such materials are peer reviewed and may be re‐organized for online delivery, but are not copy‐edited or typeset. Technical support issues arising from supporting information (other than missing files) should be addressed to the authors.

Supporting Information
