# Supplementary material for: N‐(Anilinoethyl)amide Melatonergic Ligands with Improved Water Solubility and Metabolic Stability
Source: ChemMedChem. 2021 Jul 26;16(19):3071–82. doi: 10.1002/cmdc.202100405 (PMC8518537; doi:10.1002/cmdc.202100405)
Supplement: Supplementary file 1 — Supporting Information [file CMDC-16-3071-s001.pdf]

ChemMedChem

Supporting Information

N-(Anilinoethyl)amide Melatonergic Ligands with Improved Water Solubility and Metabolic Stability

Francesca Ferlenghi, Michele Mari,* Gabriella Gobbi, Gian Marco Elisi, Marco Mor, Silvia Rivara, Federica Vacondio, Silvia Bartolucci, Annalida Bedini, Fabiola Fanini, and Gilberto Spadoni

Table of contents

Content Page

1. ^{1}H NMR and ^{13}C NMR spectra of target compounds S2

2. HPLC purity of target compounds S13

3. Equilibration protocol for MD simulations of $MT_{1/2}$ receptor-ligand complexes with compound **14** S 20

1. ^{1}H NMR and ^{13}C NMR spectra of target compounds

1-{2-[(3-Methoxyphenyl)phenylamino]ethyl}urea (**6a**).

^{1}H NMR (400 MHz, $CDCl_3$)

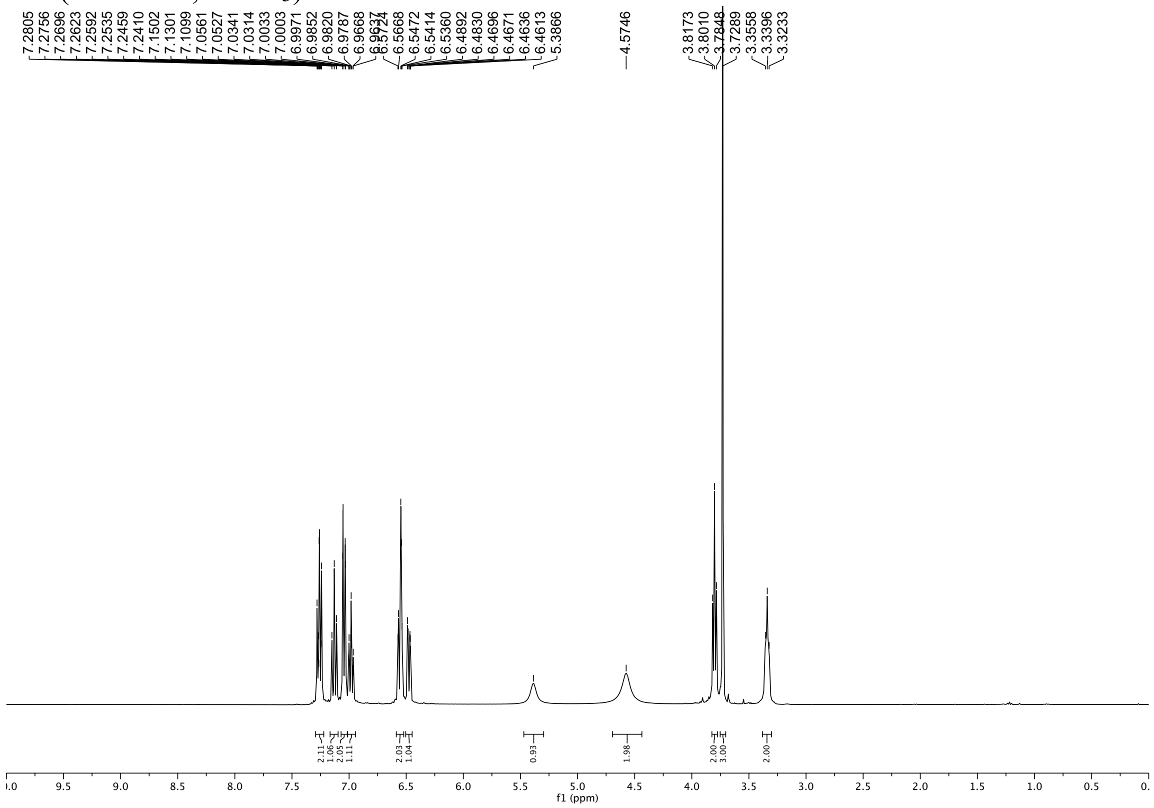

^{13}C NMR (100 MHz, $CDCl_3$)

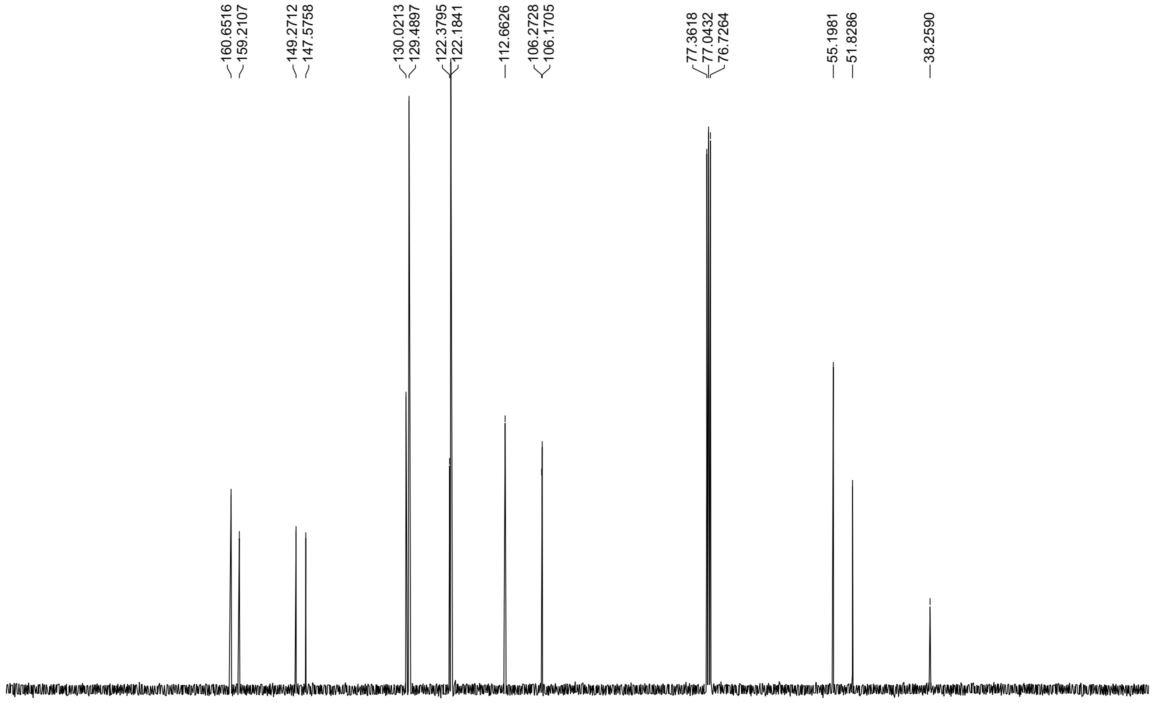

1-Ethyl-3-{2-[(3-methoxyphenyl)phenylamino]ethyl}urea (**6b**)

^{1}H NMR (400 MHz, $CDCl_3$)

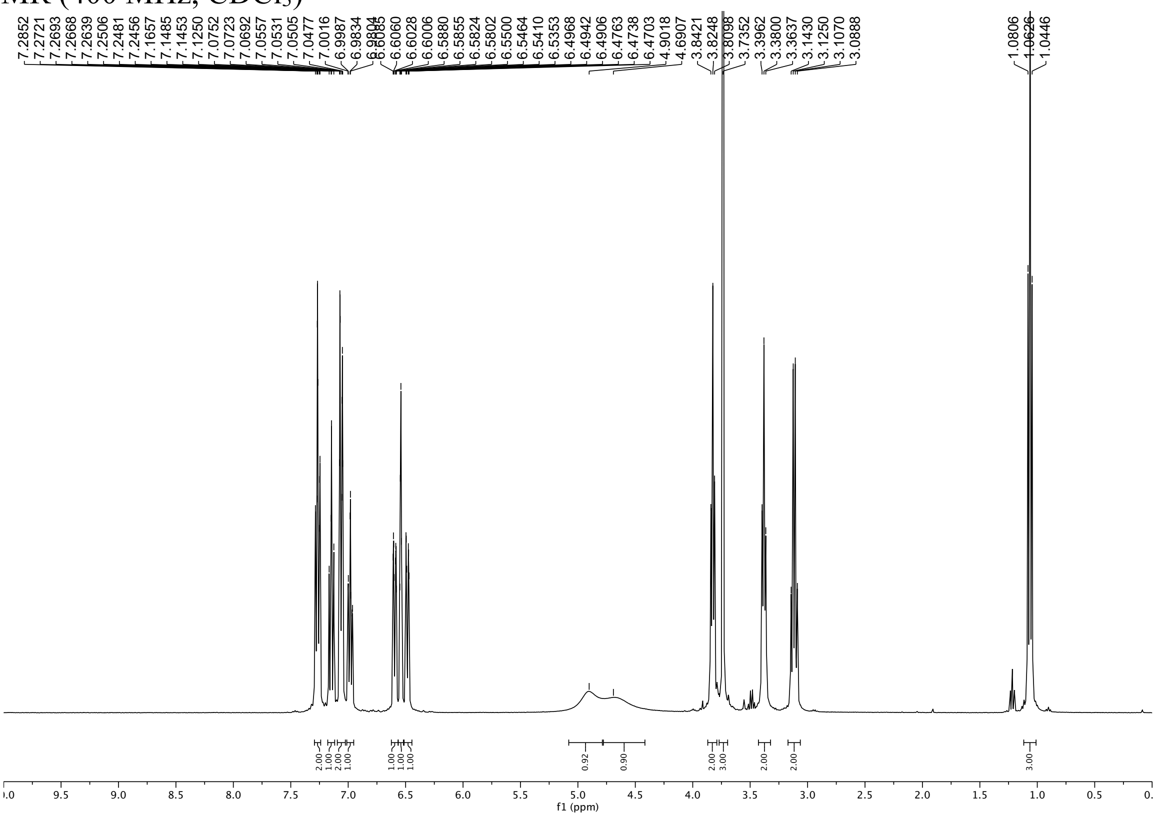

^{13}C NMR (100 MHz, $CDCl_3$)

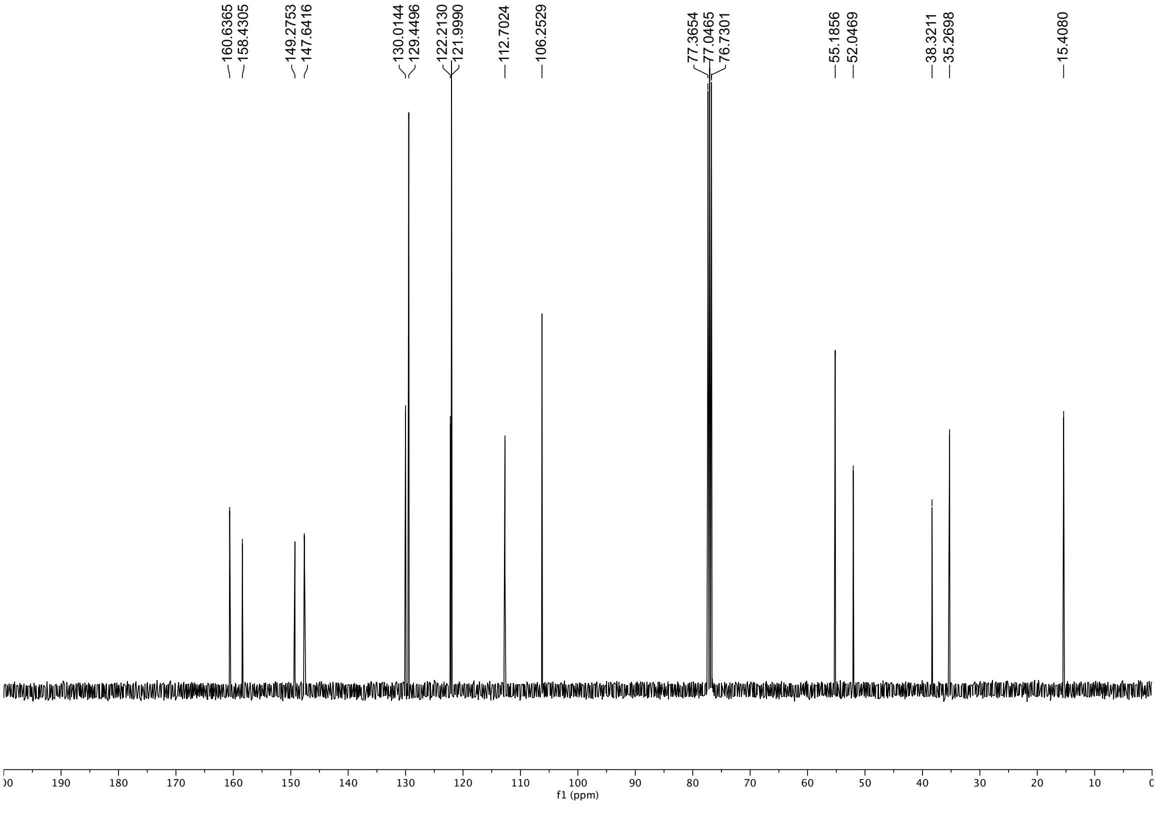

1-{2-[(3-Methoxyphenyl)phenylamino]ethyl}-3-propylurea (**6c**)

^{1}H NMR (400 MHz, $CDCl_3$)

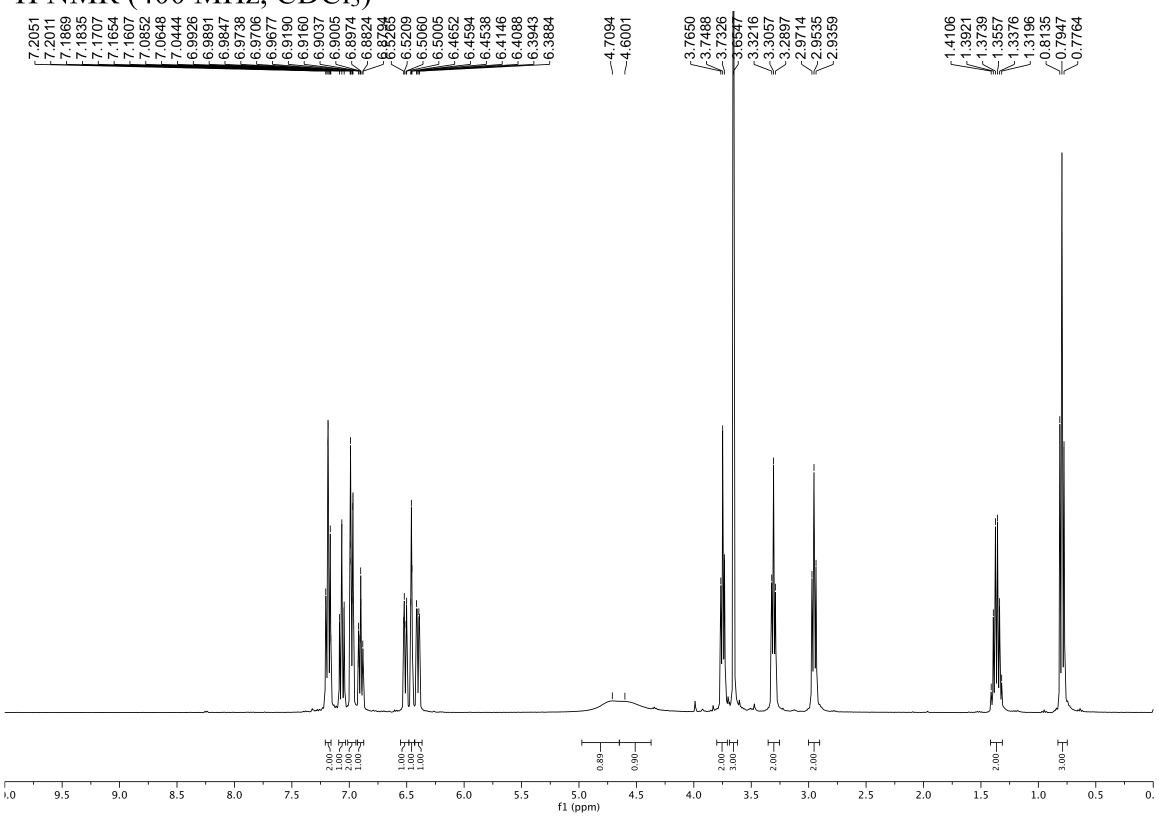

^{13}C NMR (100 MHz, $CDCl_3$)

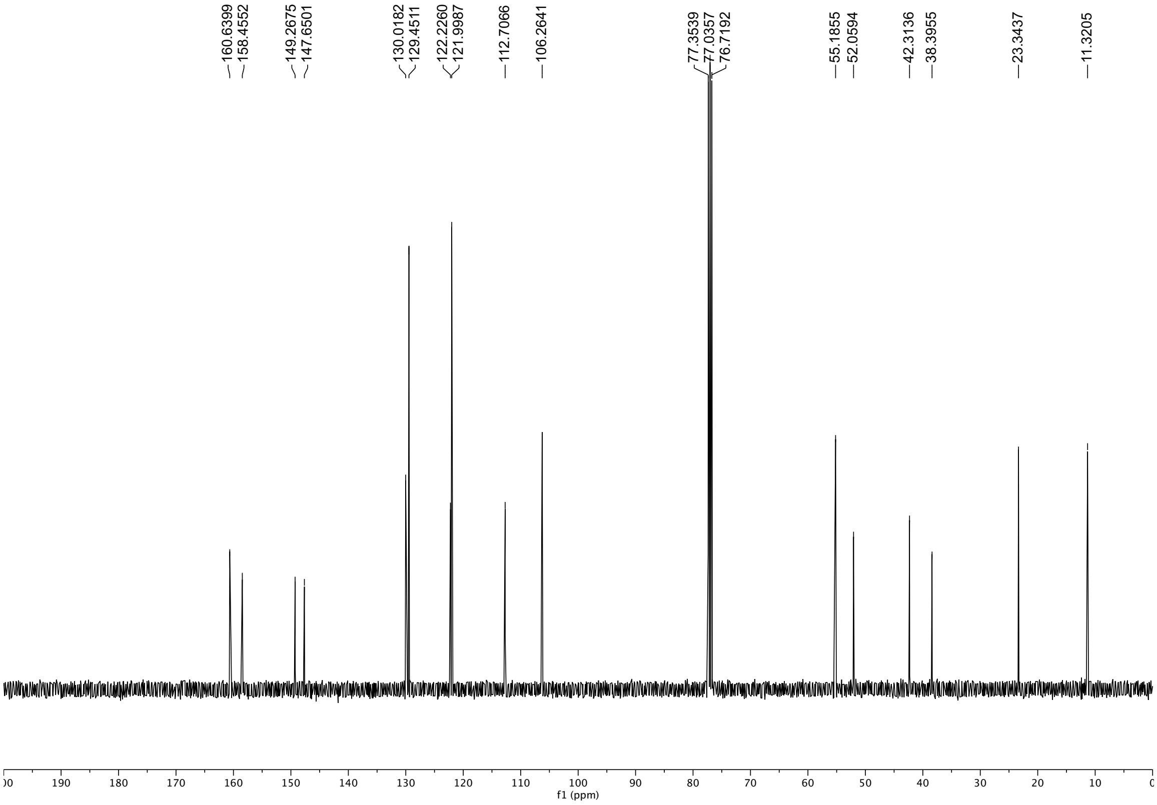

N-(2-{[3-(Difluoromethyl)phenyl](4-fluorophenyl)amino}ethyl)acetamide (**10**)

^{1}H NMR (400 MHz, $CDCl_3$)

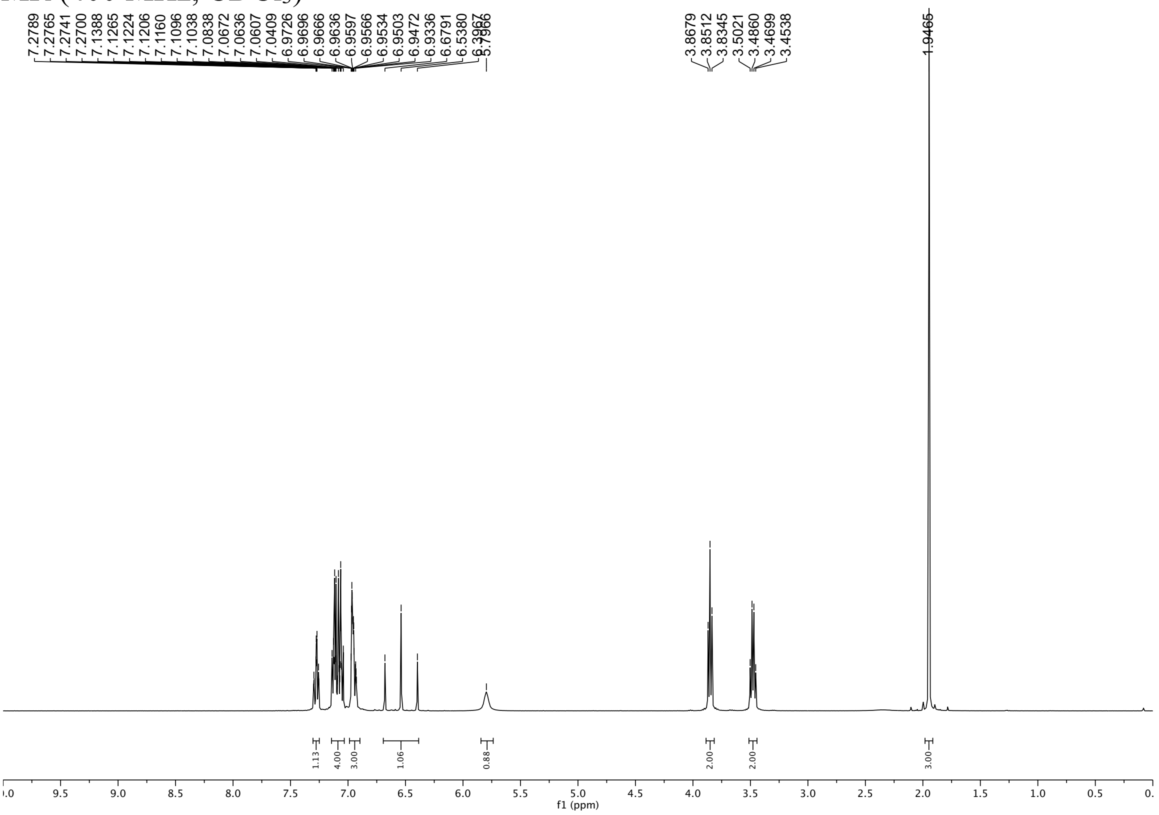

^{13}C NMR (100 MHz, $CDCl_3$)

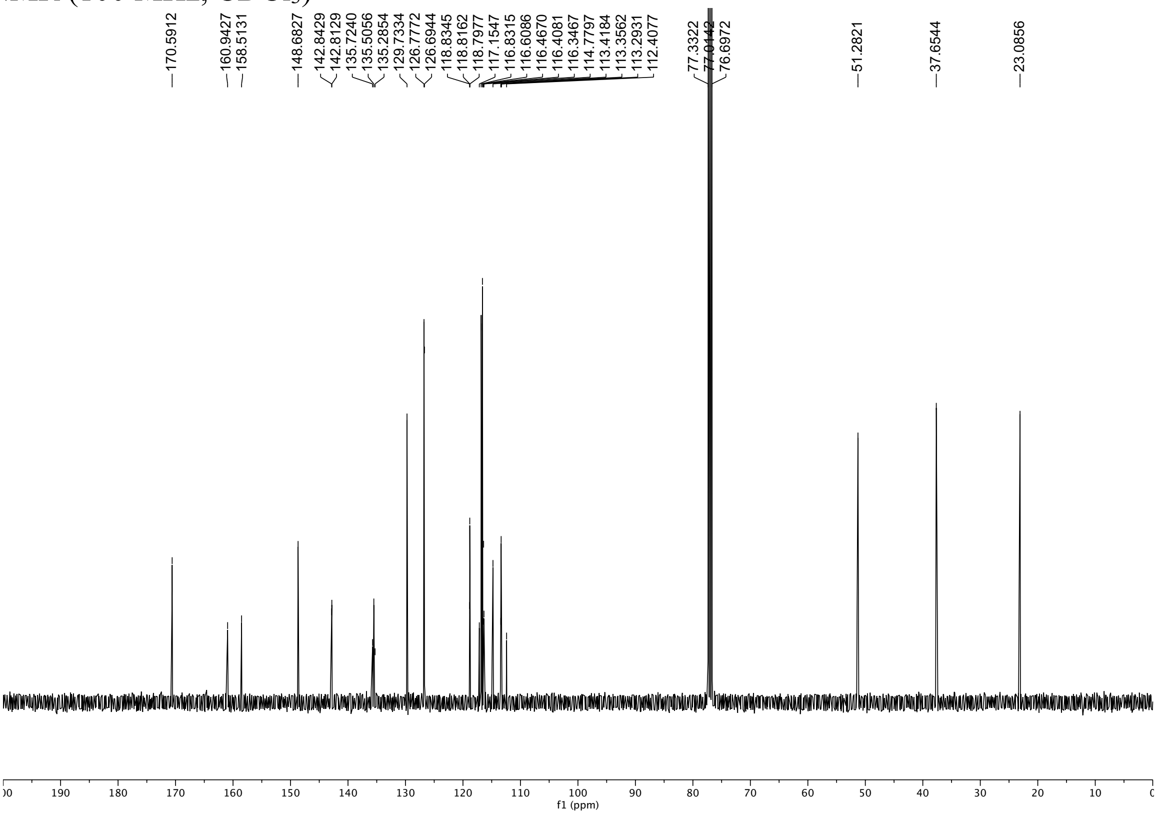

N-{2-[(3-Methoxyphenyl)(thiophen-3-yl)amino]ethyl}acetamide (**11**)

^{1}H NMR (400 MHz, $CDCl_3$)

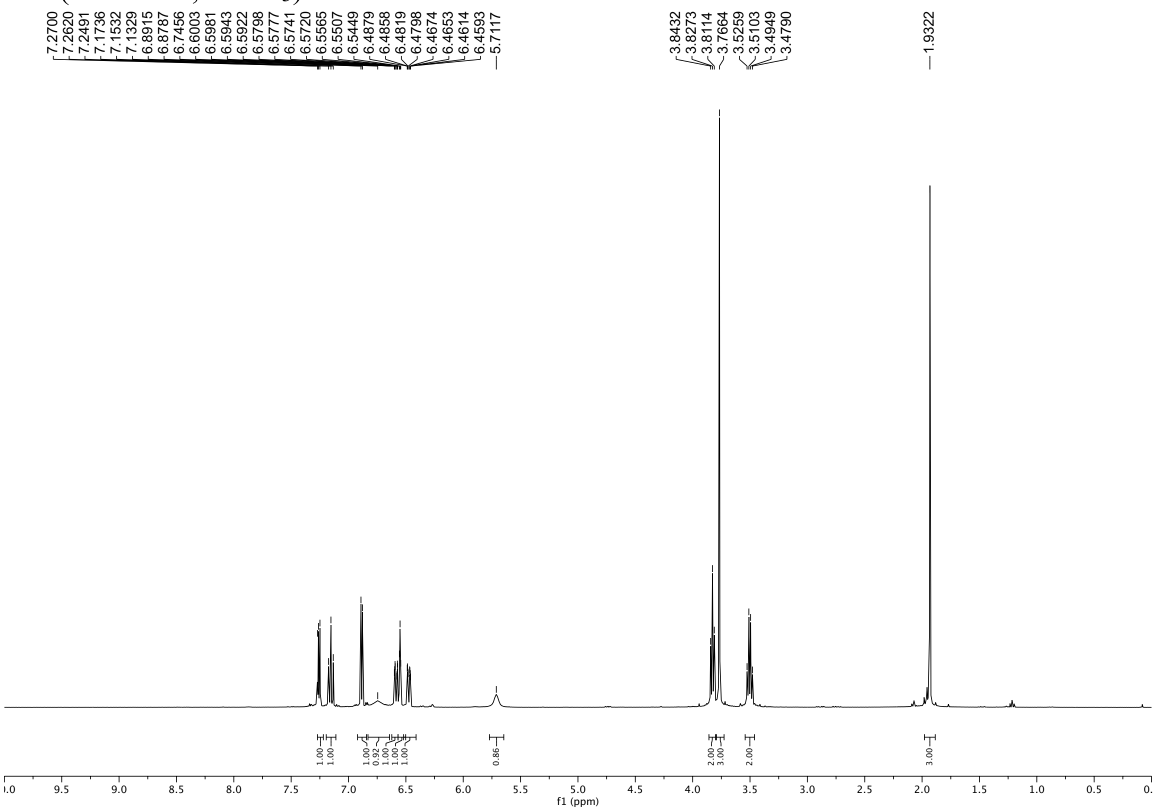

^{13}C NMR (100 MHz, $CDCl_3$)

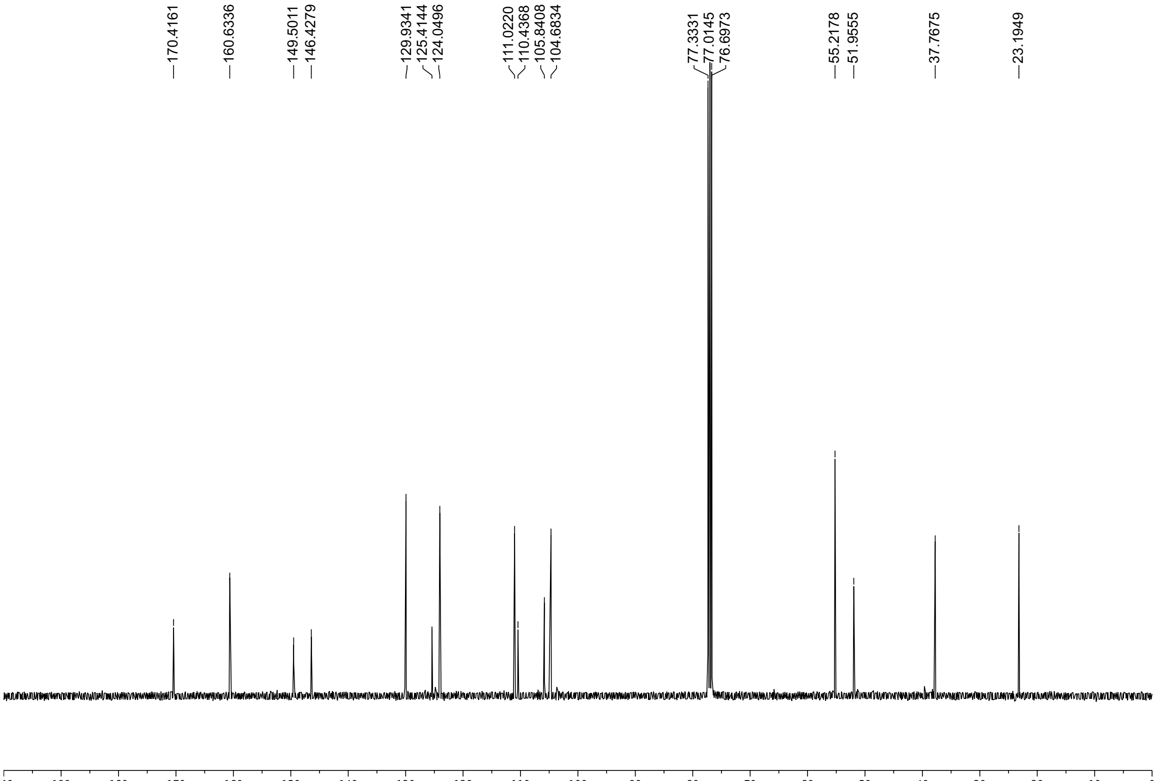

N-{2-[(3-Methoxyphenyl)(phenethyl)amino]ethyl}acetamide (**12**)

^{1}H NMR (400 MHz, $CDCl_3$)

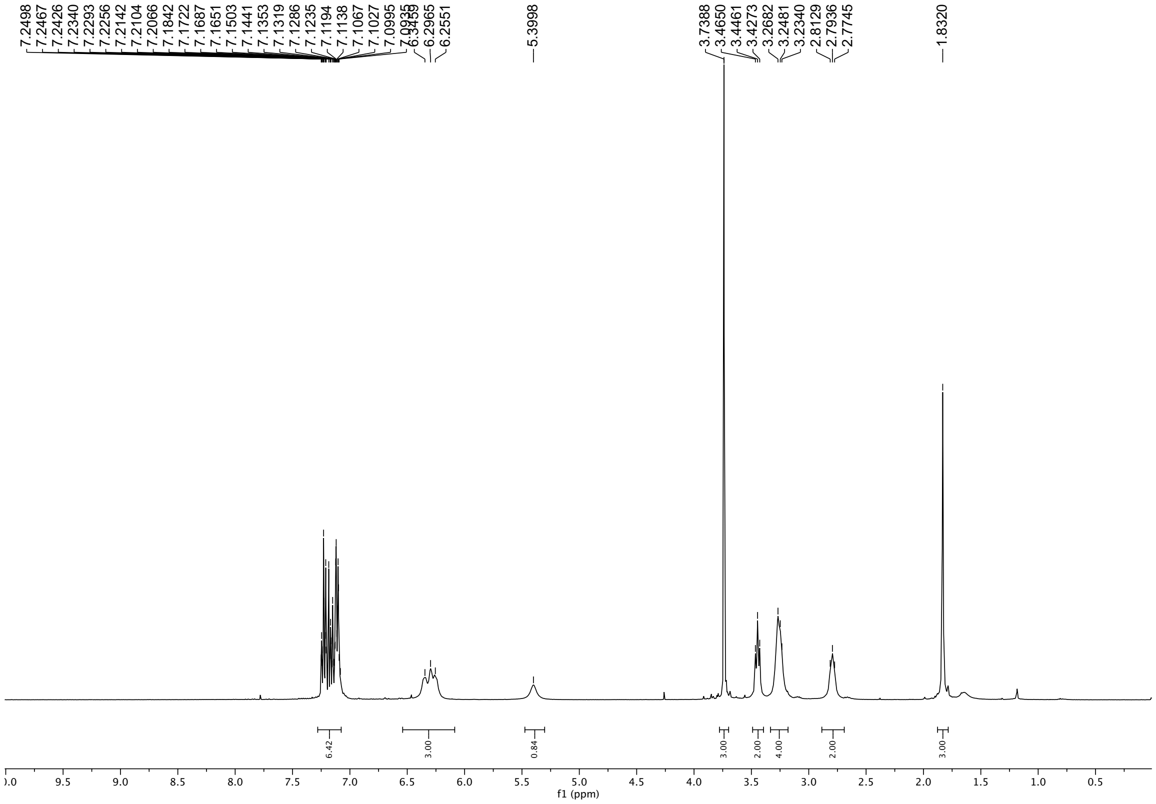

^{13}C NMR (100 MHz, $CDCl_3$)

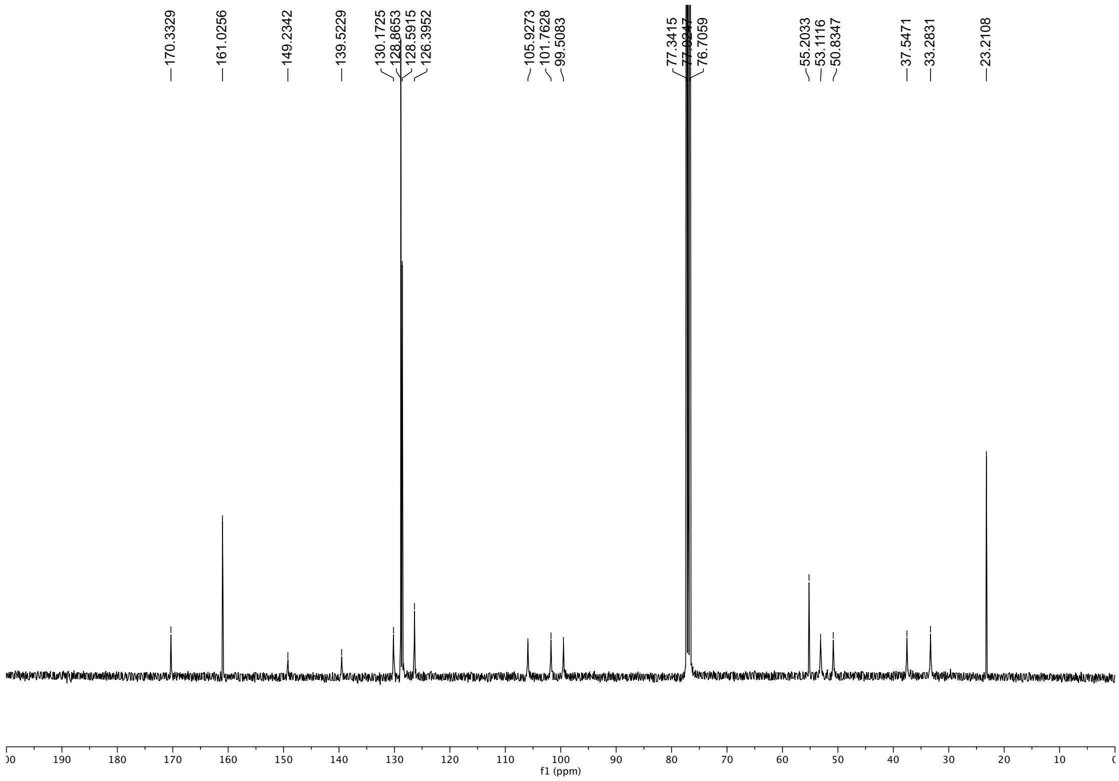

N-{2-[(3-Methoxyphenyl)(pyridine-4-yl)amino]ethyl}acetamide (**13**)

^{1}H NMR (400 MHz, $CDCl_3$)

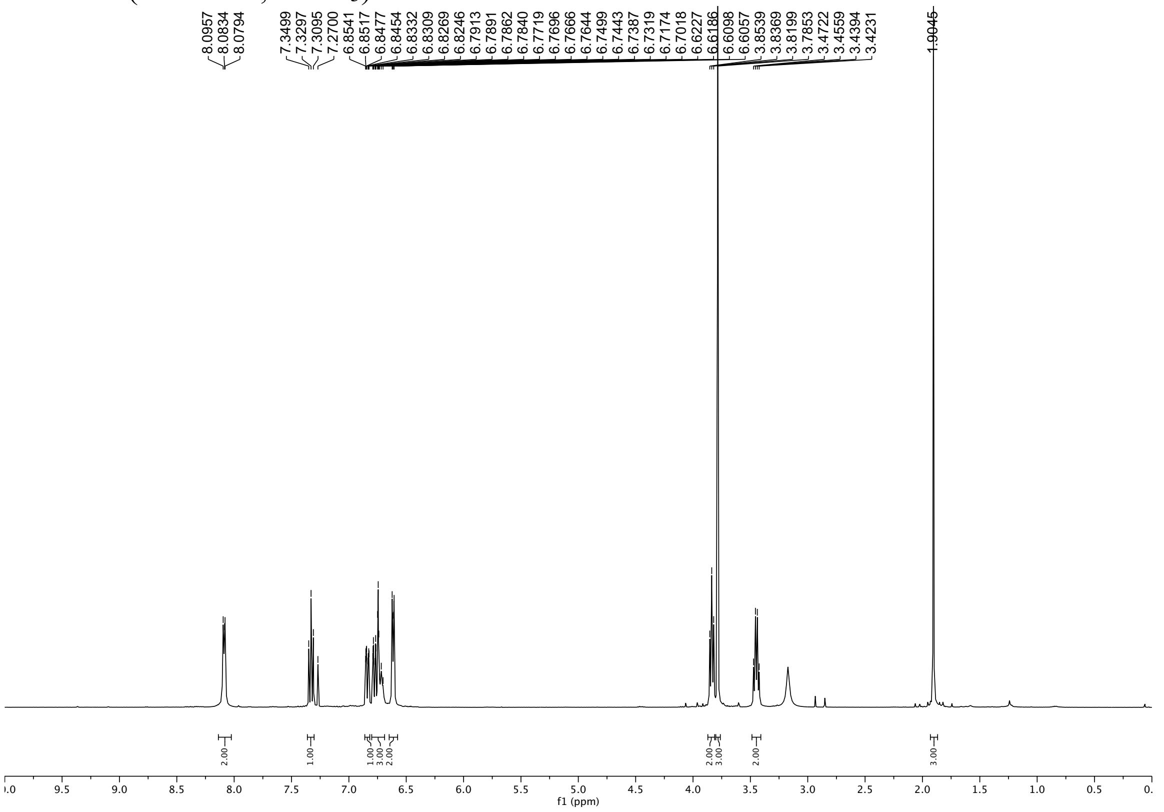

^{13}C NMR (100 MHz, $CDCl_3$)

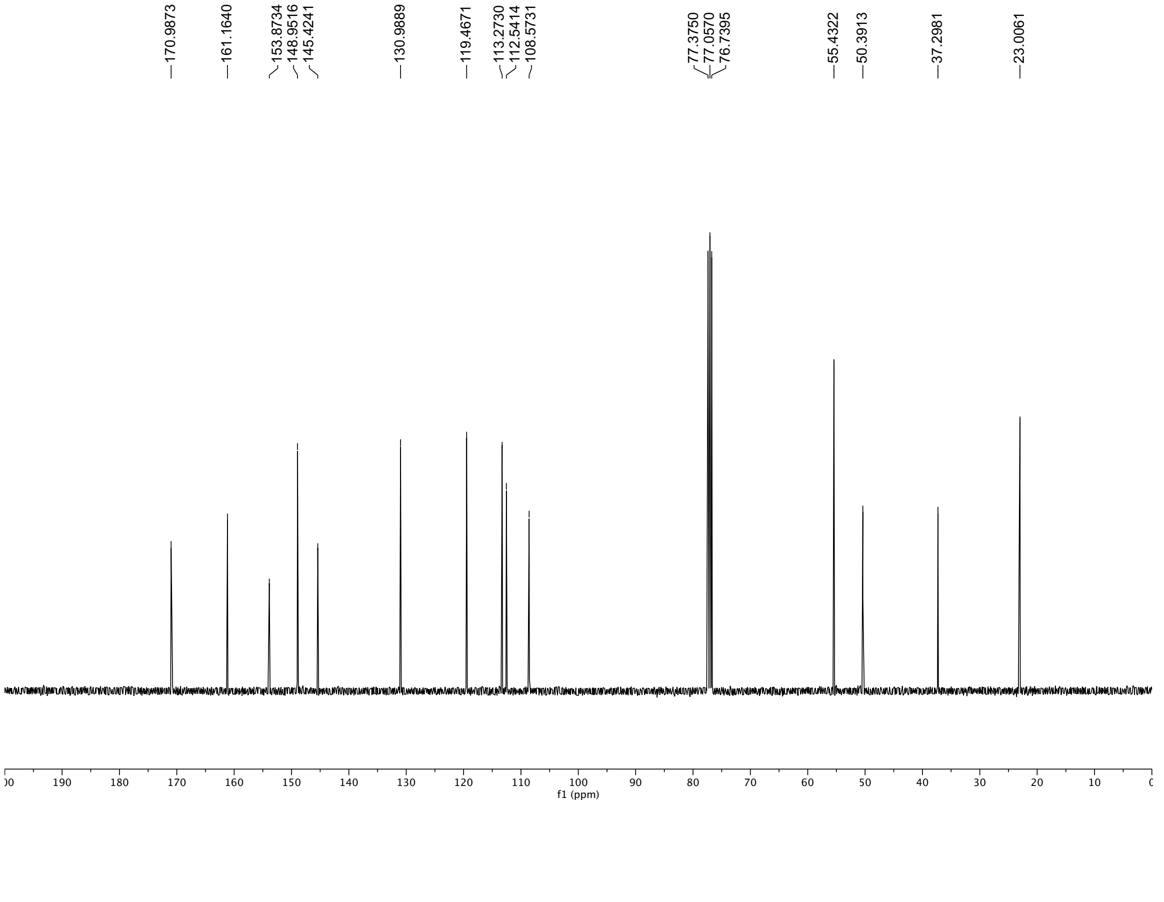

N-{2-[(3-Hydroxymethylphenyl)(3-methoxyphenyl)amino]ethyl} acetamide (**14**)

^{1}H NMR (400 MHz, $CDCl_3$)

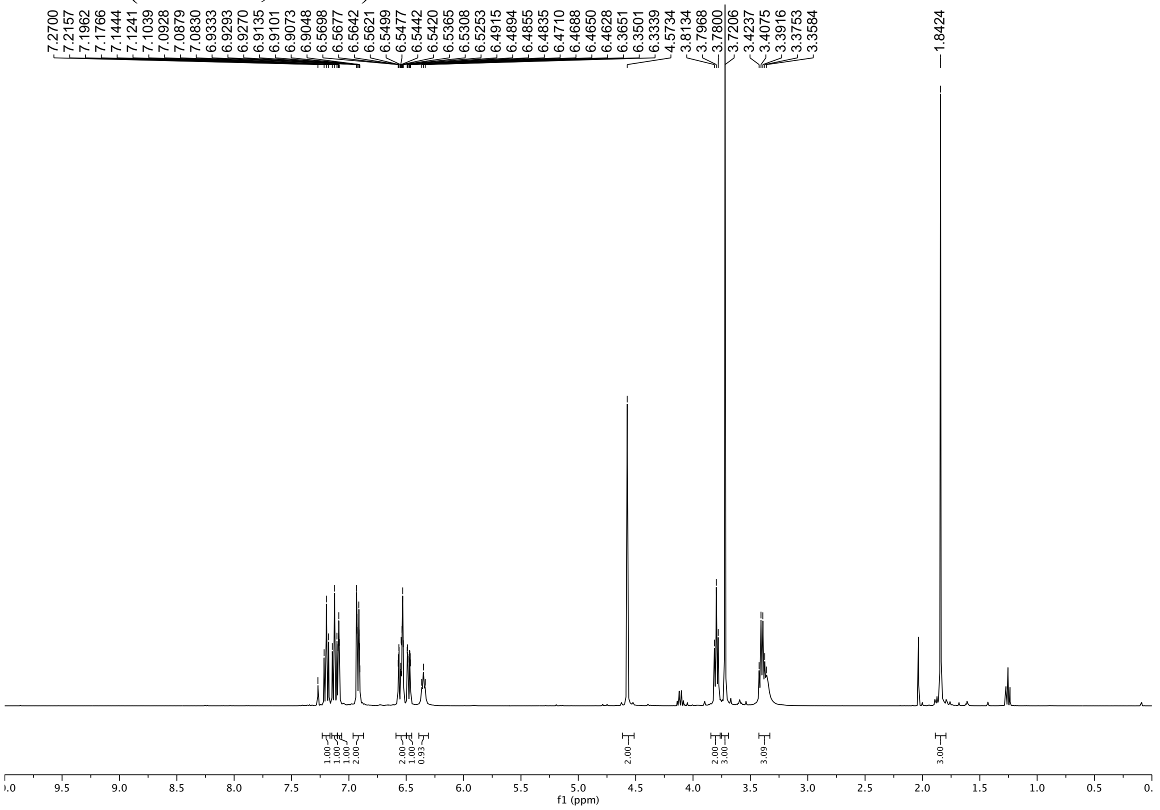

^{13}C NMR (100 MHz, $CDCl_3$)

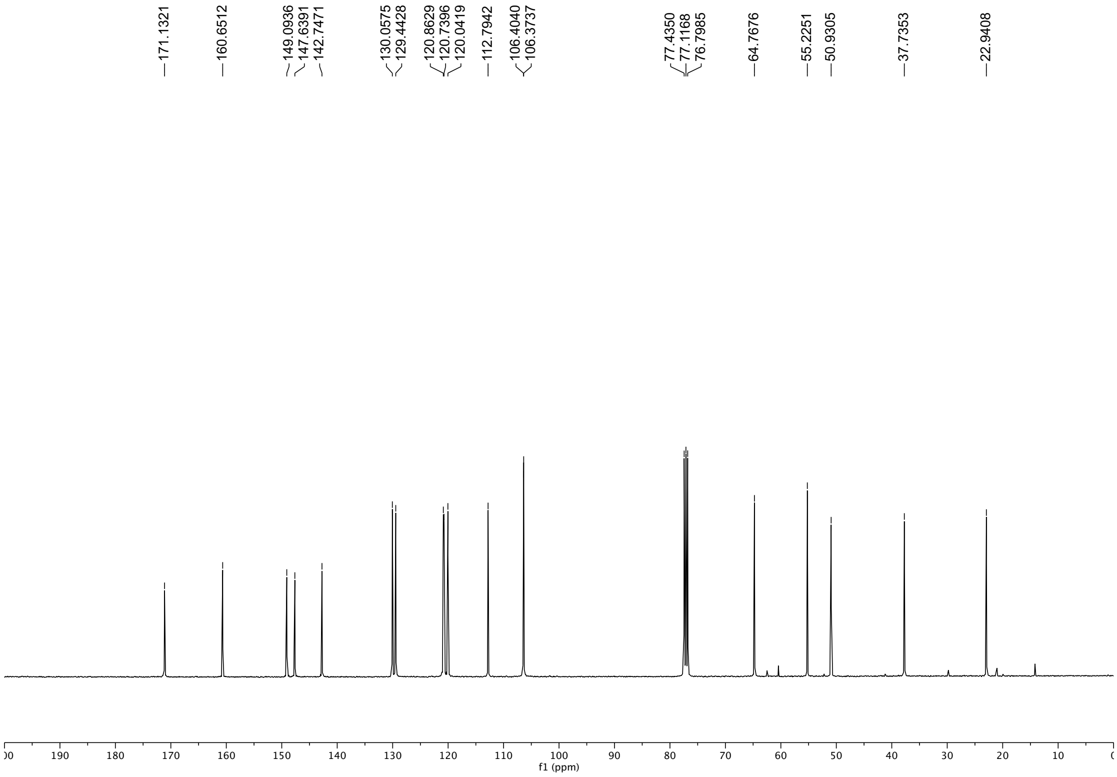

N-{2-[(3-Hydroxyphenyl)(3-methoxyphenyl)amino]ethyl} acetamide (**15**)

^{1}H NMR (400 MHz, $CDCl_3$)

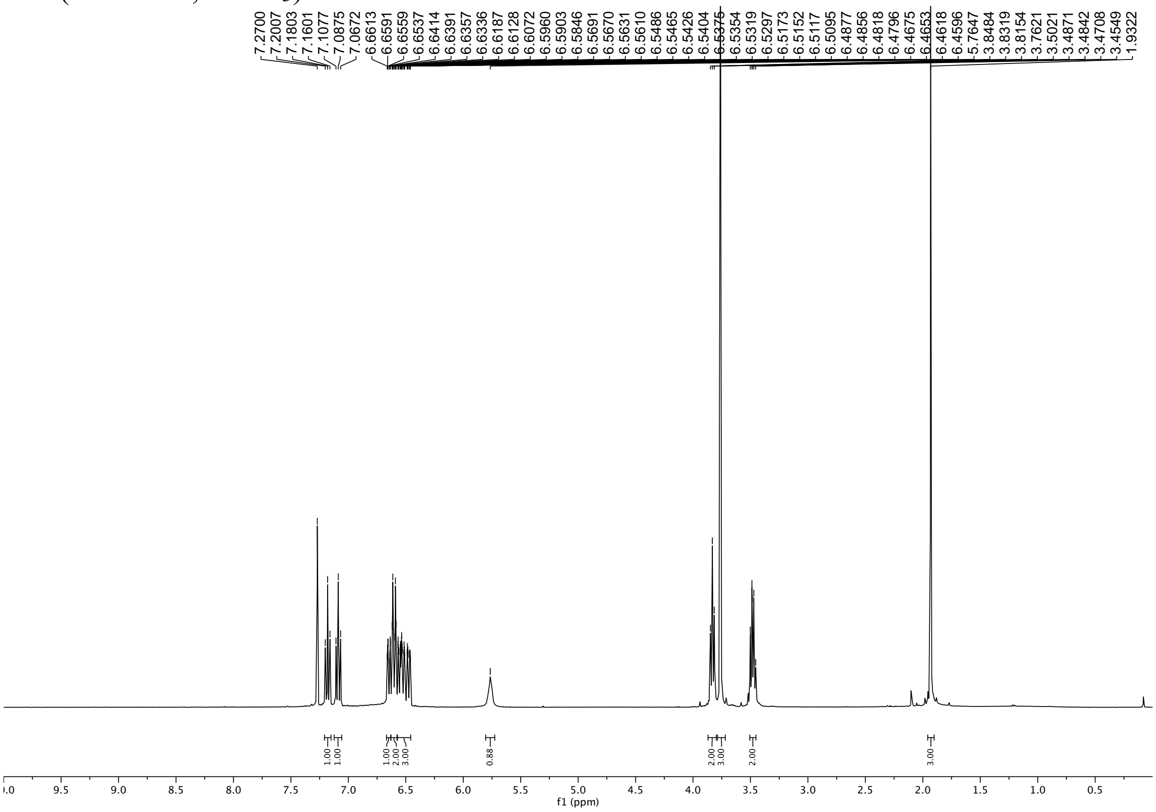

^{13}C NMR (100 MHz, $CDCl_3$)

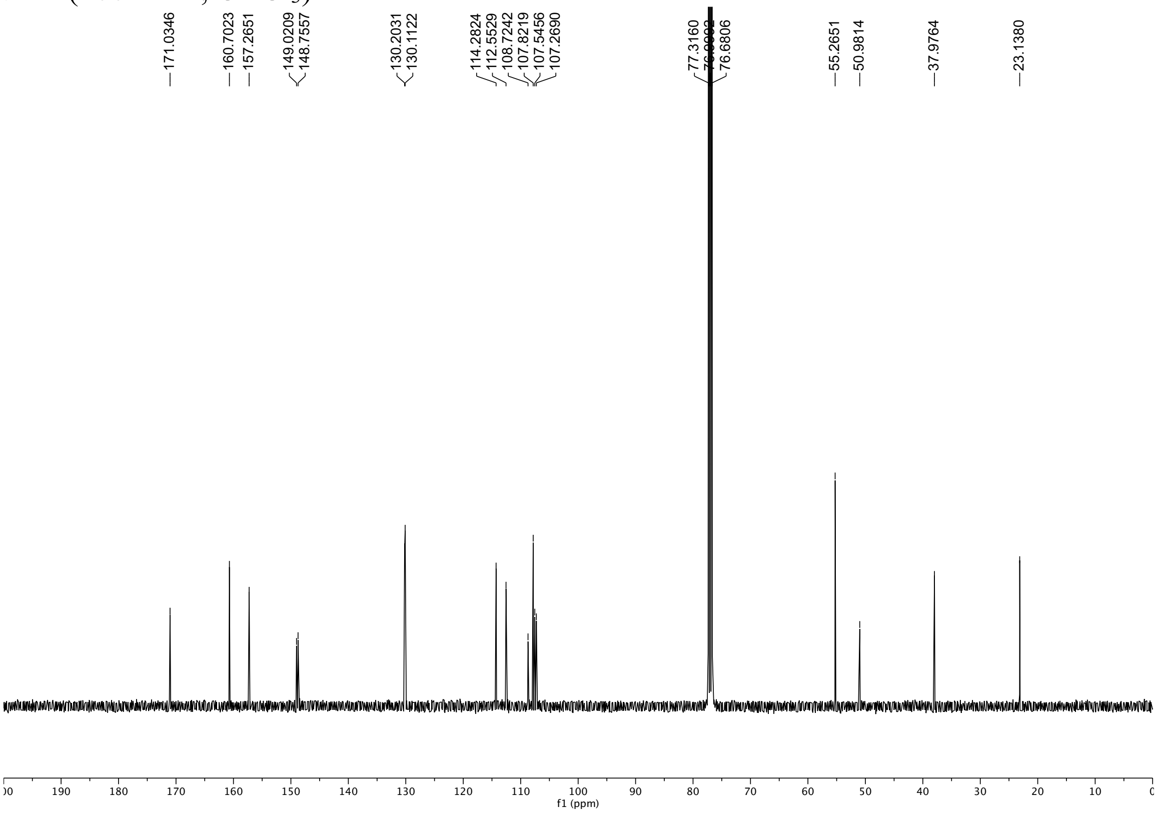

N-{2-[(4-Methoxy-[1,1'-biphenyl]-2-yl)(methylamino)]ethyl}acetamide (**18**)

^{1}H NMR (400 MHz, $CDCl_3$)

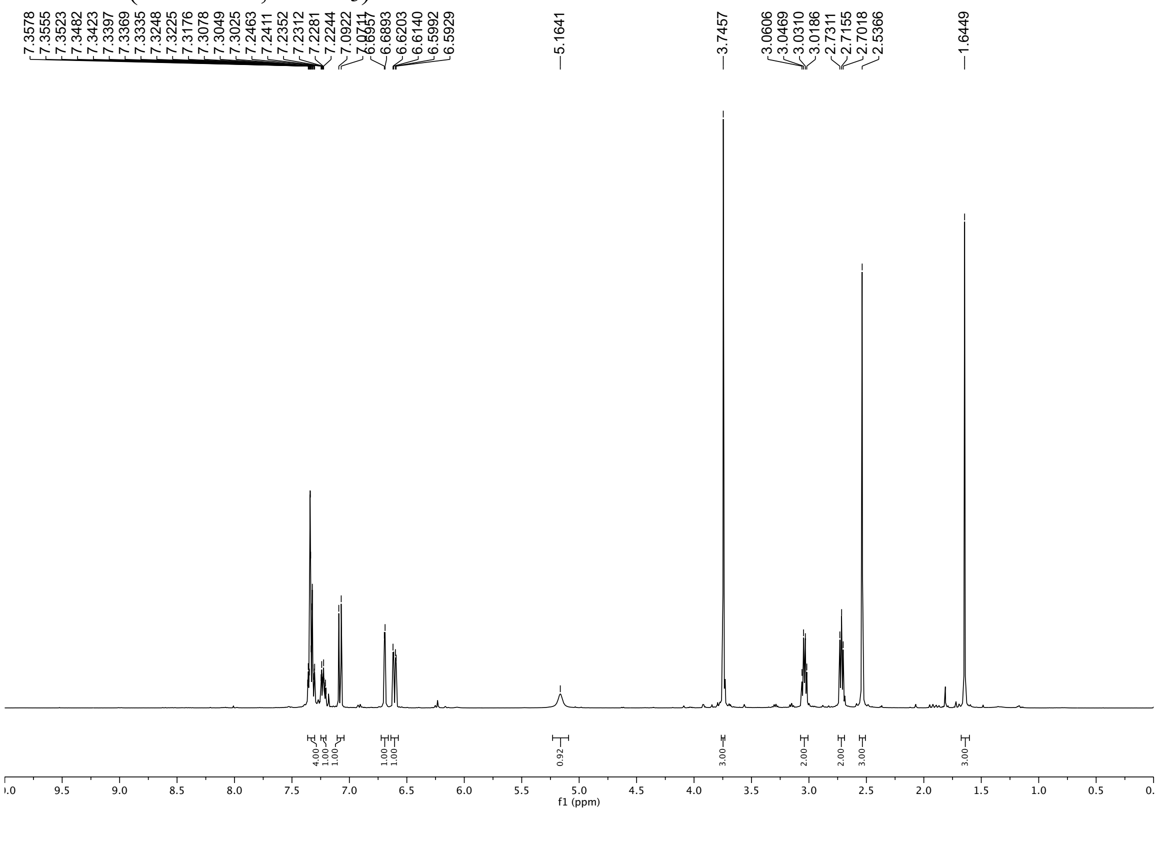

^{13}C NMR (100 MHz, $CDCl_3$)

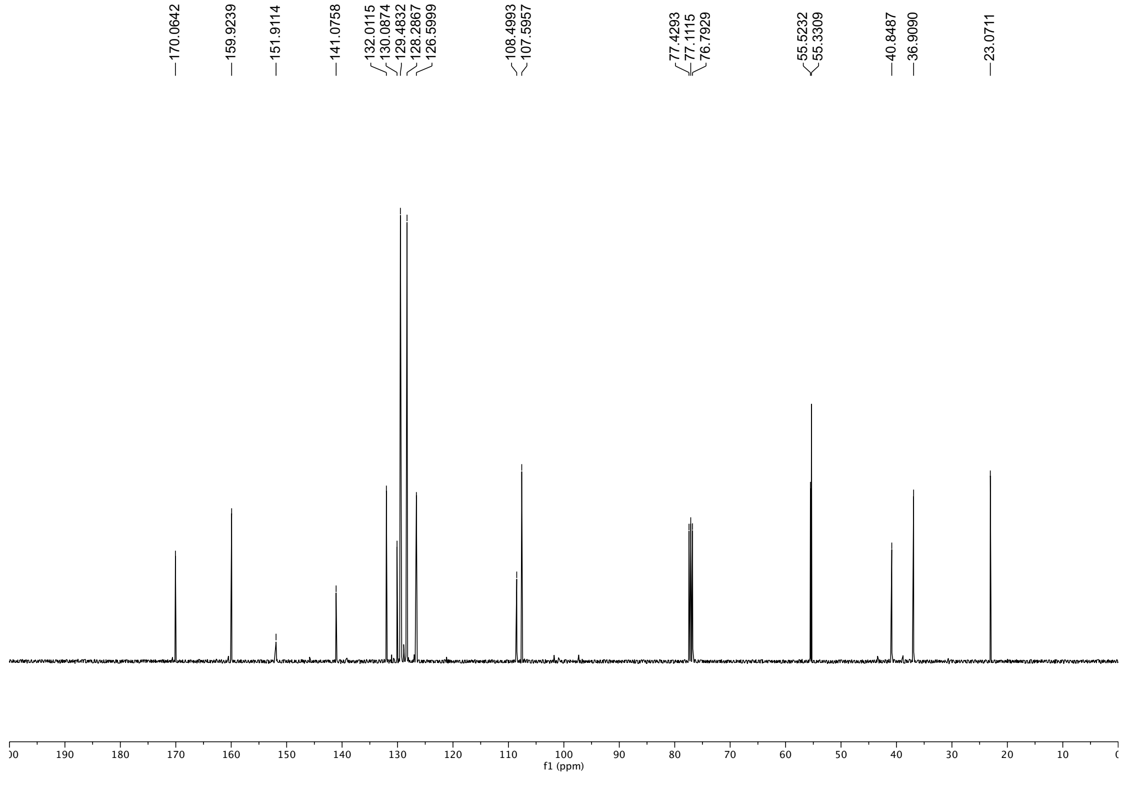

N-{2-[(2-Bromo-5-methoxyphenyl)methylamino]ethyl}acetamide (**19**)

^{1}H NMR (400 MHz, $CDCl_3$)

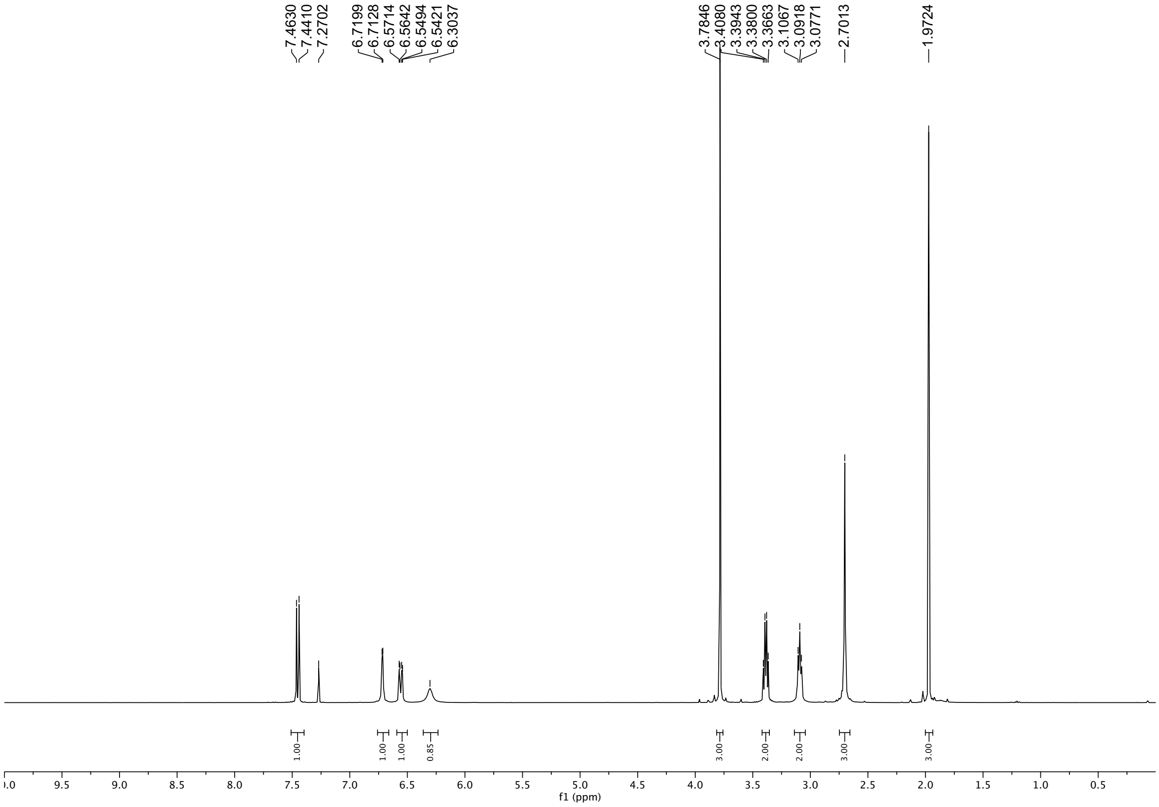

^{13}C NMR (100 MHz, $CDCl_3$)

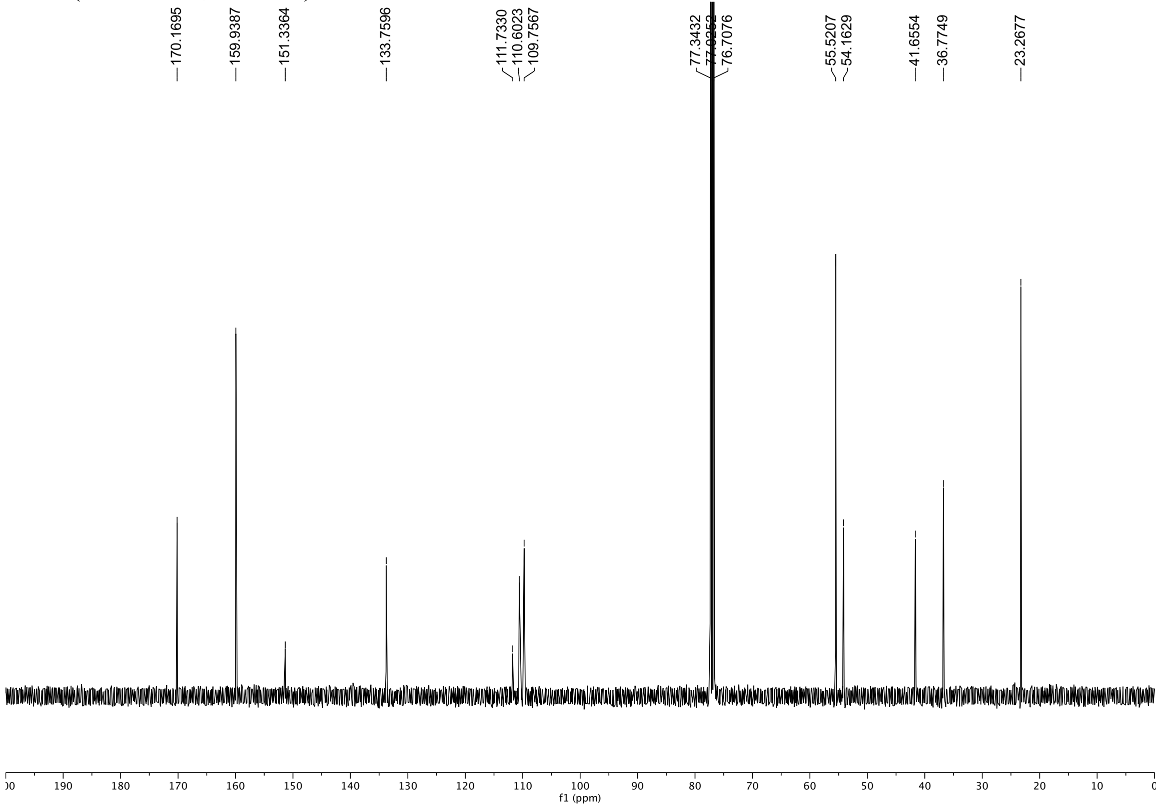

2. HPLC purity of target compounds

The purity of all tested compounds was determined by HPLC analysis and was greater than 95%. Analyses were performed on a Waters HPLC/UV/MS system (separation module Alliance HT2795, Photo Diode Array Detector 2996, mass detector Micromass ZQ; software: MassLynx 4.1). Separation was achieved using Gemini® C6-Phenyl column (1500 mm × 4.6 mm i.d., 5-µm particle size).

Linear gradient of 0.1% formic acid aqueous solution and acetonitrile 40/60 to 100% acetonitrile for 8 minutes then 100% acetonitrile for 2 minutes.

HPLC settings were as follows: flow rate, 0.8 mL/min; injection volume, 10.0 µL; and detector wavelength, 254 nm.

Table S1

Compound	Rt	Purity % (λ 254 nm)
**6a**	2.77	98.7
**6b**	3.35	99.3
**6c**	3.70	98.2
**10**	3.37	100
**11**	3.10	100
**12**	3.43	100
**13**	1.23	98.6
**14**	2.42	100
**15**	2.55	100
**18**	3.12	97.3
**19**	2.62	100

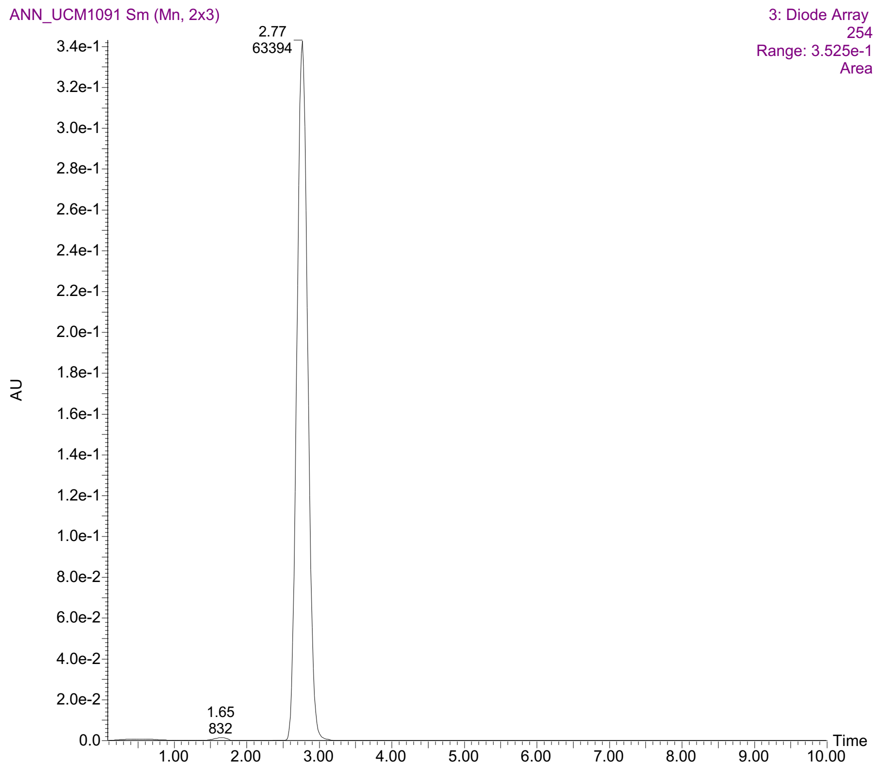

HPLC trace of compound 6a.

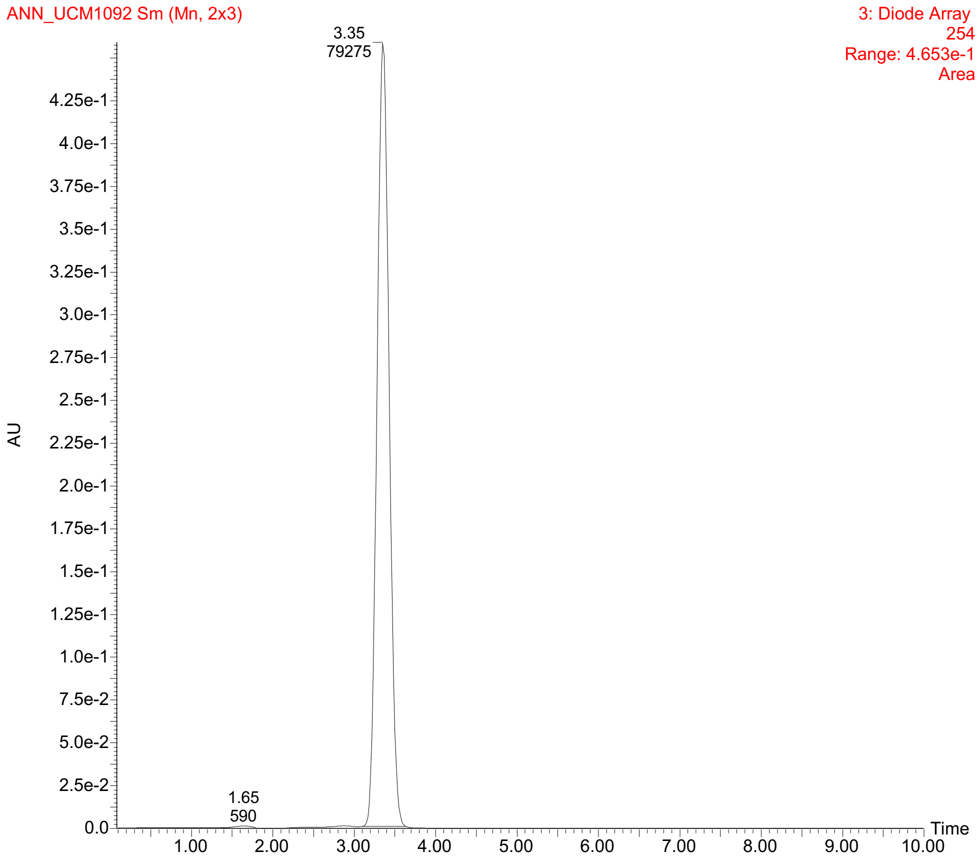

HPLC trace of compound 6b.

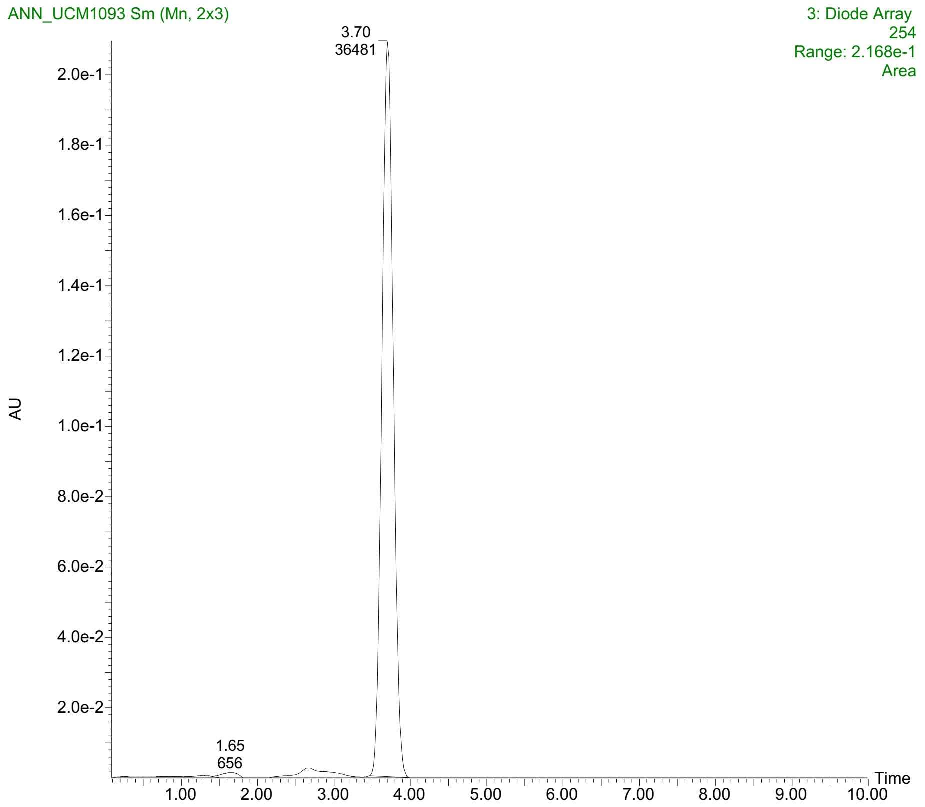

HPLC trace of compound 6c.

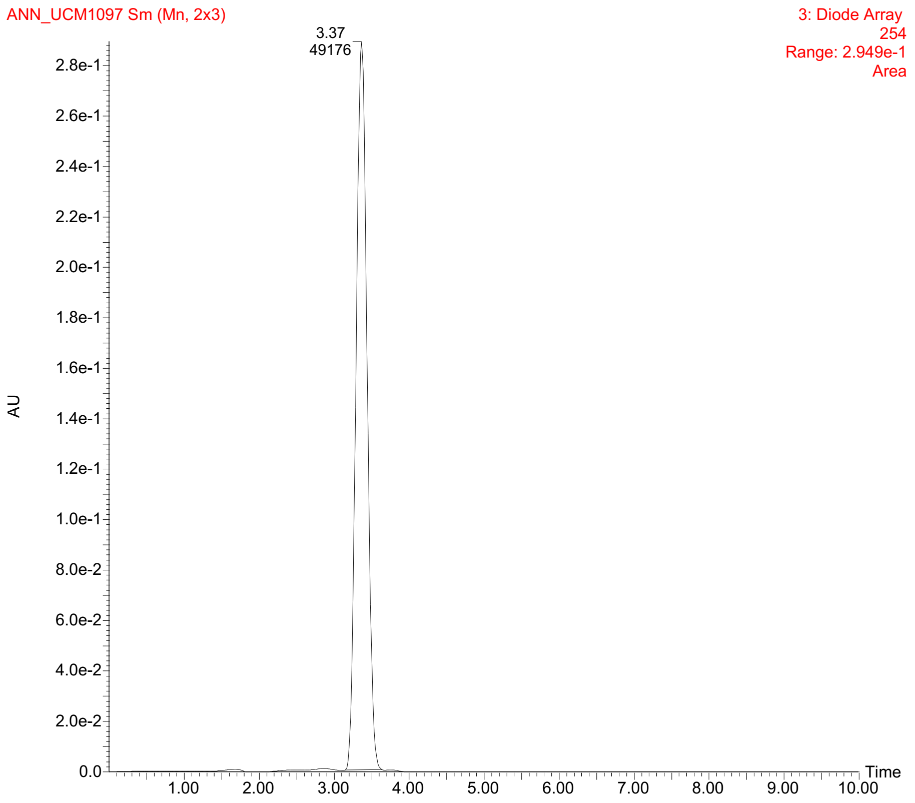

HPLC trace of compound 10.

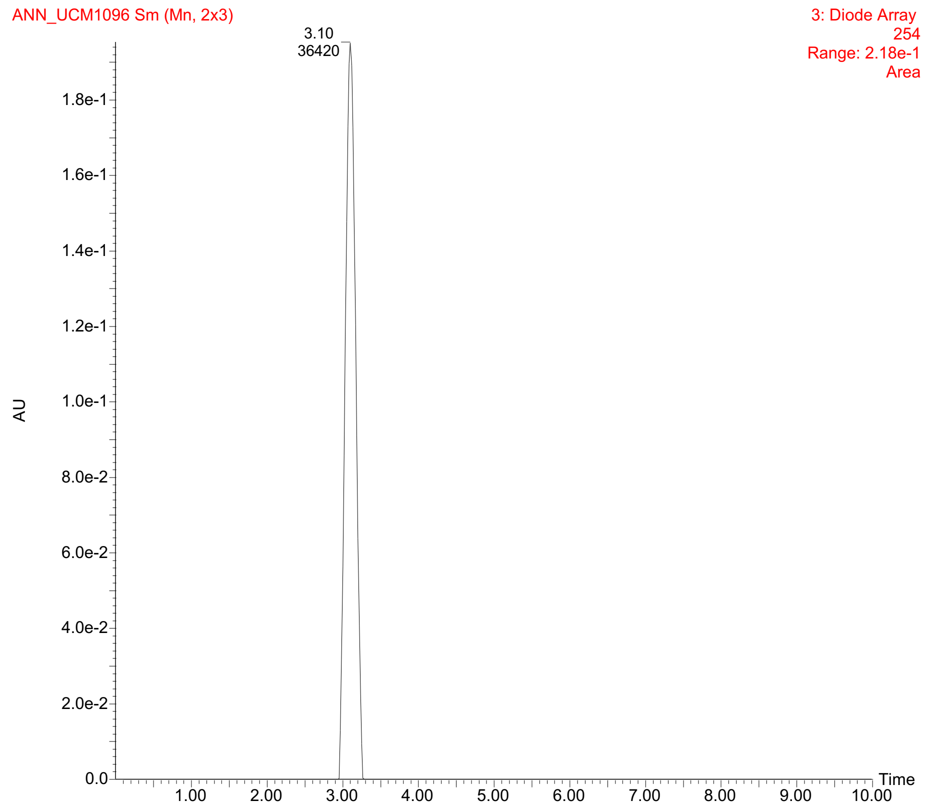

HPLC trace of compound 11.

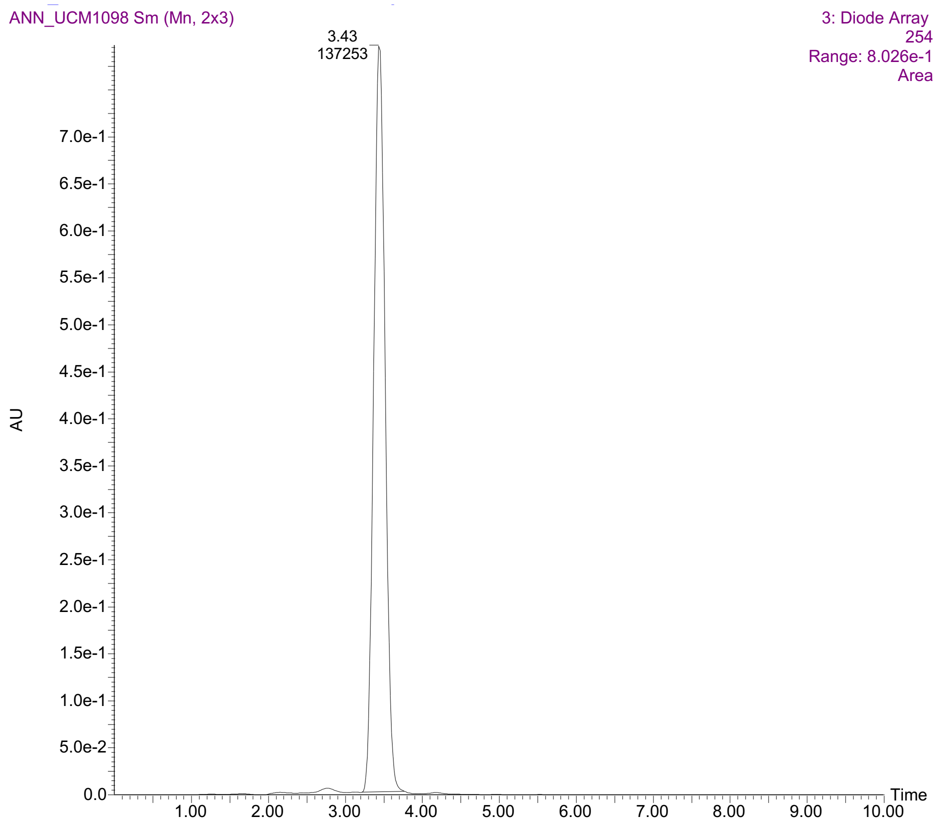

HPLC trace of compound 12.

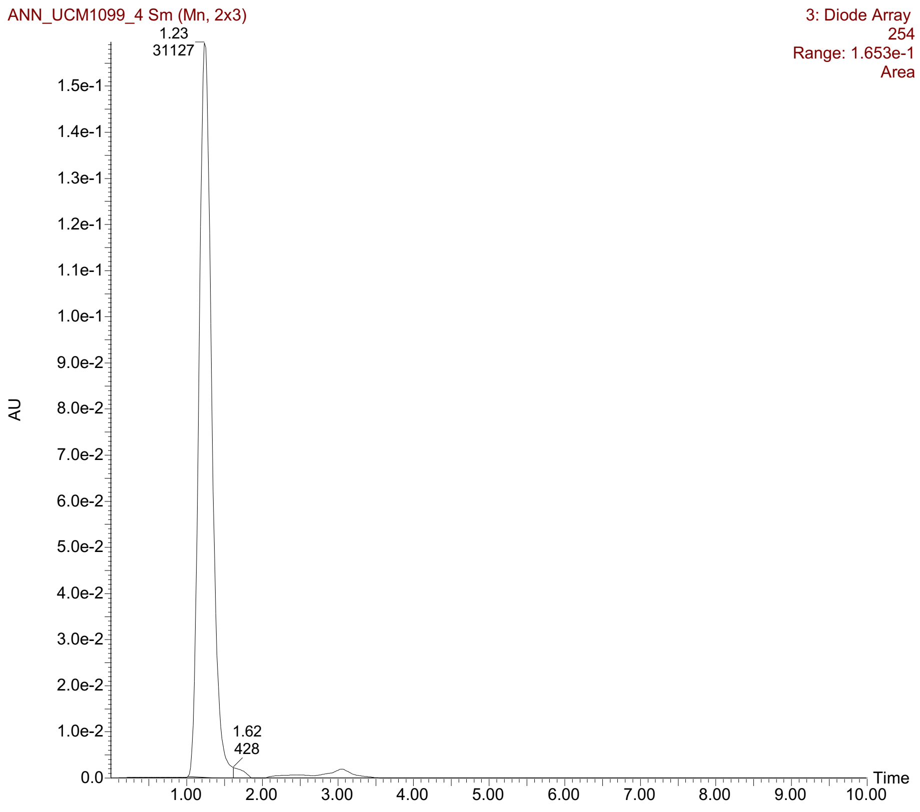

HPLC trace of compound 13.

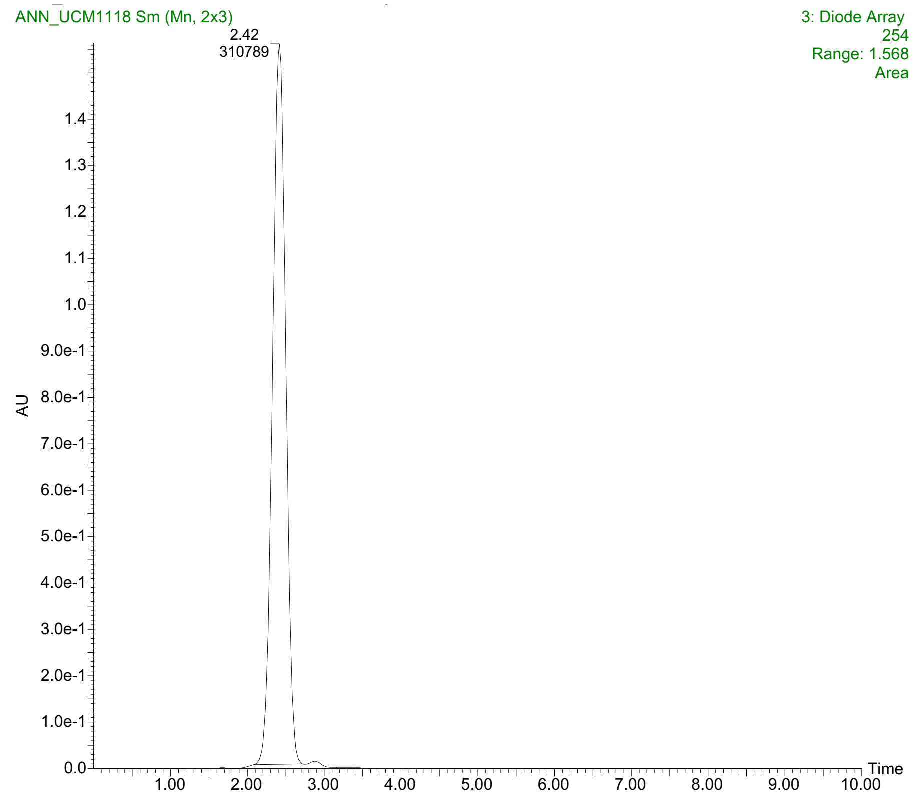

HPLC trace of compound 14.

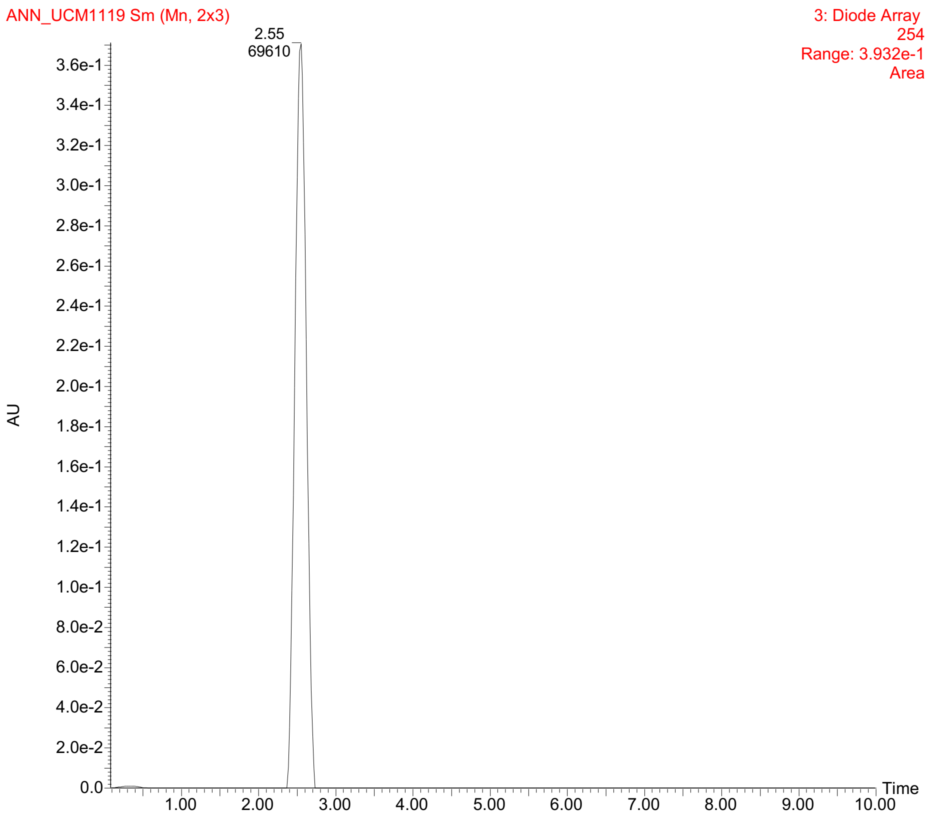

HPLC trace of compound 15.

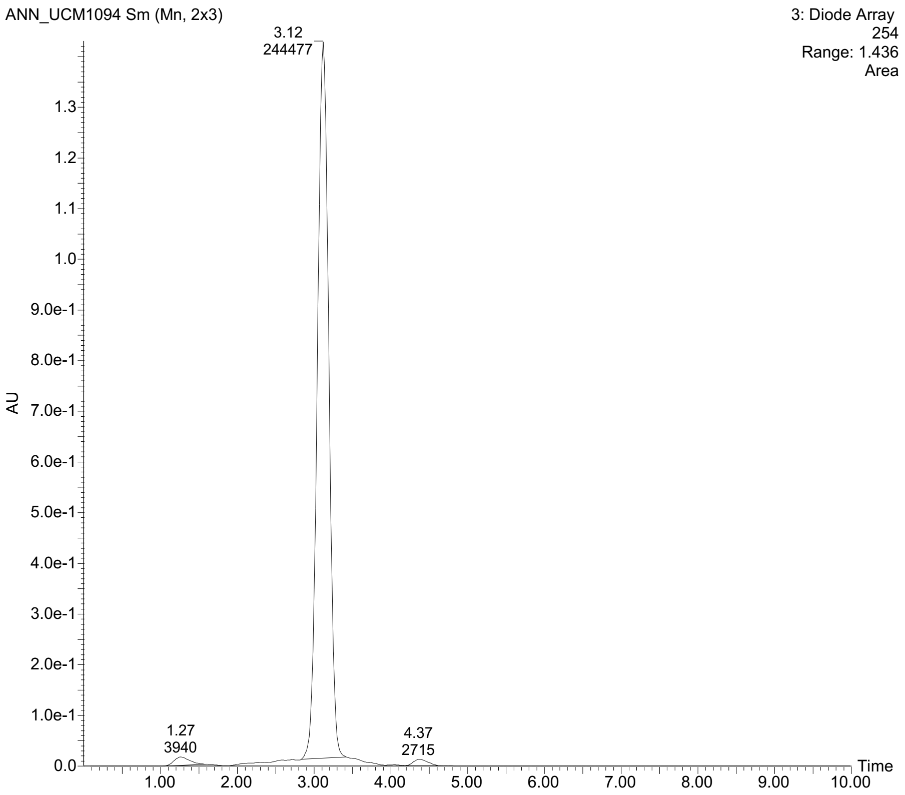

HPLC trace of compound 18

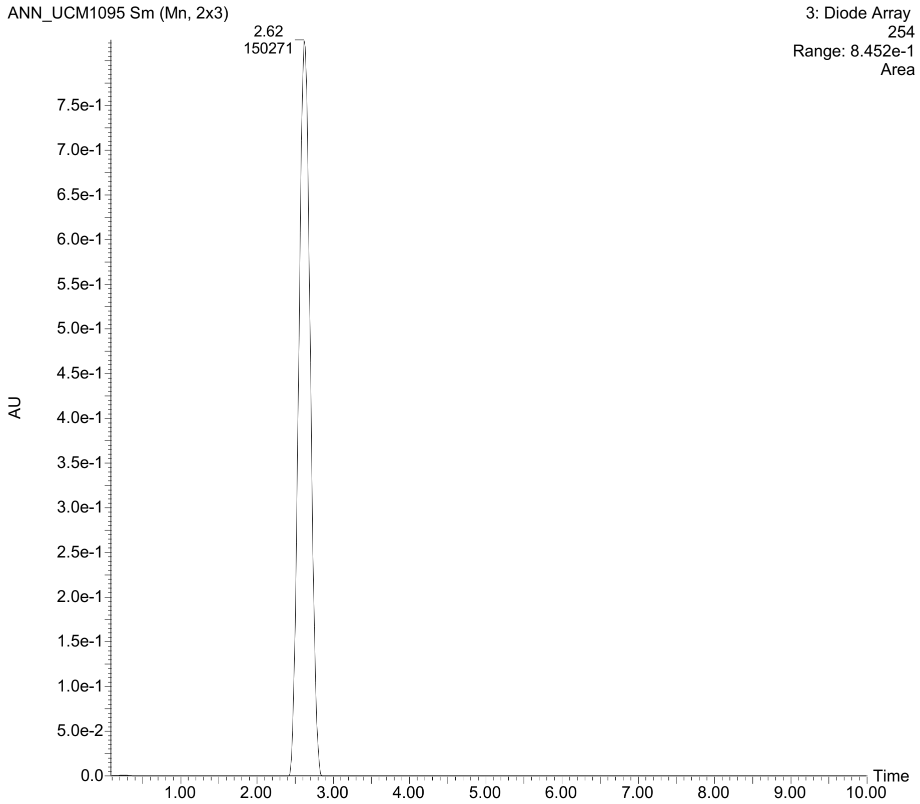

HPLC trace of compound 19.

3. Equilibration protocol for MD simulations of $MT_{1/2}$ receptor complexes with compound 14

The equilibration protocol consists of a variation of the default relaxation procedure implemented in Desmond 5.4 for protein-membrane systems with additional NVT steps with gradual release of restraint.

1. 50 ps of Brownian dynamics in NVT ensemble with 1 ps timestep (3 ps for long-range electrostatics) for at 10 K with a force constant of 50 kcal·mol^{-1}·Å^{-2} on the solute heavy atoms;

2. 200 ps of Brownian dynamics in NPT ensemble with 1 ps timestep (3 ps for long-range electrostatics) at 100 K with a force constant of 20 kcal·mol^{-1}·Å^{-2} on the solute heavy atoms. A directional restraint on Z-axis was applied to membrane heavy atoms with a force constant of 5 kcal·mol^{-1}·Å^{-2};

3. 250 ps in NPγT ensemble[1] with a 1 ps timestep (3 ps for long-range electrostatics) at 100 K. Solute heavy atoms were restrained with a force constant of 10 kcal·mol^{-1}·Å^{-2}. A gaussian-shaped barrier potential was placed at the membrane-solvent interface to enhance the lipid stabilization.[2] A directional restraint on Z-axis was applied to membrane polar heads heteroatoms with a force constant of 2 kcal·mol^{-1}·Å^{-2};

4. 500 ps of linear gradient heating in NPγT ensemble[1] from 100 K to 300 K with the same timesteps used during production (2 ps and 6 ps for long-range electrostatics) and a force constant of 10 kcal·mol^{-1}·Å^{-2} on the solute heavy atoms. A gaussian-shaped barrier potential was placed at the membrane-solvent interface to enhance the lipid stabilization.[2] A directional restraint on Z-axis was still applied to choline heads polar heteroatoms with a force constant of 2 kcal·mol^{-1}·Å^{-2};

5. 4.0 ns in NVT ensemble[3,4] with temperature fixed at 300 K, coupled with a constant of 1 ps. This stage is further divided in steps with gradual release of restraints and timesteps as in production:
  - 1.0 ns with restraints on backbone and ligand heavy atoms of 5 kcal·mol^{-1}·Å^{-2};
  - 1.0 ns with restraints on alpha carbons and ligand heavy atoms of 5 kcal·mol^{-1}·Å^{-2};
  - 1.0 ns with restraints on alpha carbons of 2.5 kcal·mol^{-1}·Å^{-2} and ligand heavy atoms of 1 kcal·mol^{-1}·Å^{-2}. Henceforth, restraints on cap-termini backbone heavy atoms are kept as in the production phase (see main text).
  - 1.0 ns with further differentiated restraint on the alpha carbons:
    i. Helices alpha carbons are restrained with a spring constant of 2.5 kcal·mol^{-1}·Å^{-2},
    ii. Other alpha carbons have a spring constant reduced to 0.1 kcal·mol^{-1}·Å^{-2}).
    Ligand heavy atoms are kept restrained with a force constant of 0.5 kcal·mol^{-1}·Å^{-2};

6. 1.0 ns in NPγT ensemble[1] with the ligand heavy atoms restrained with a force constant of 0.1 kcal·mol^{-1}·Å^{-2}. Restraints over the alpha carbons are applied as in the production.

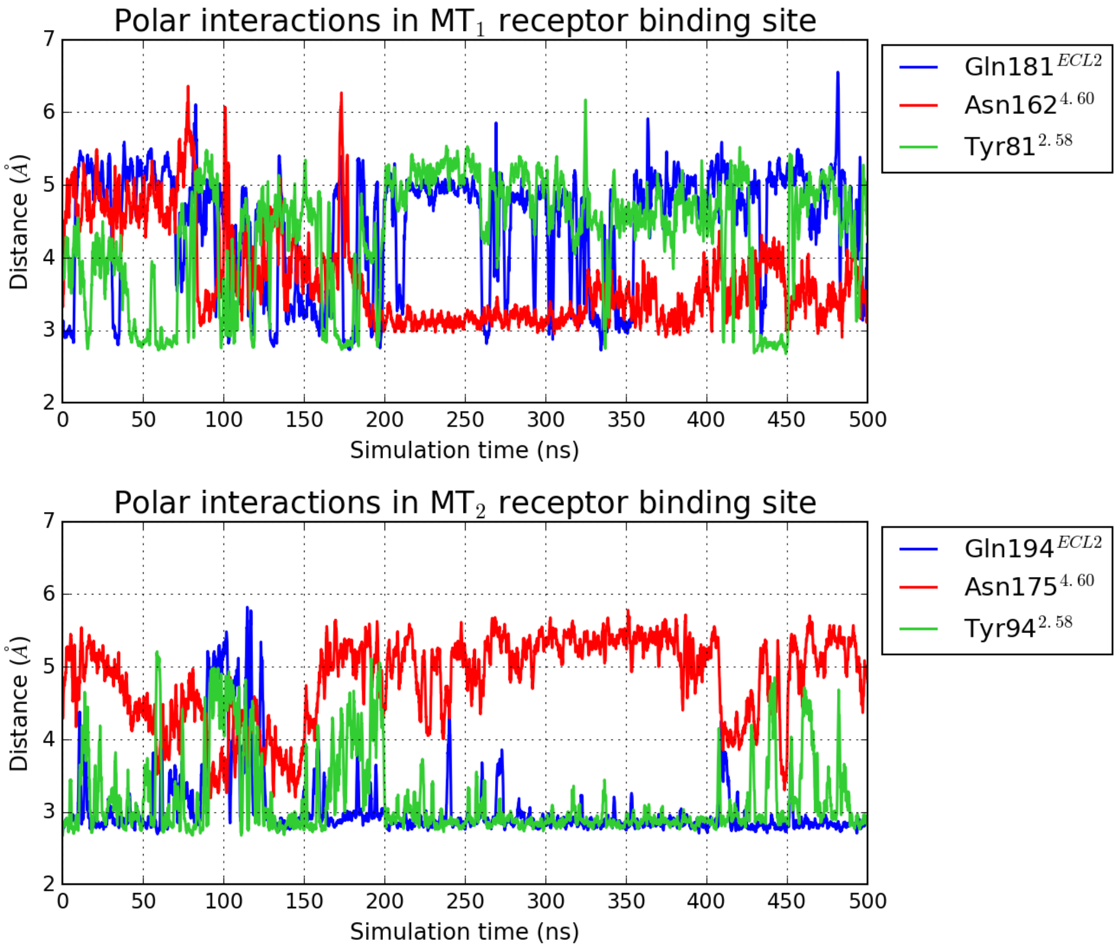

Figure S1. Polar contacts analysis of the molecular dynamics of the compound **14** in the MT_1 and MT_2 receptors' binding sites. The moving average of the distance over 1.0 ns is measured between heavy atoms involved in the hydrogen bonds: between the oxygen of the amide group and the nitrogen of the amide group of the Gln181/194^{ECL2} (blue line), between the oxygen of the methoxy group and the amide nitrogen of the Asn162/175$^{4.60}$ (red line), and between the oxygens of the hydroxymethyl moiety and the hydroxyl group of Tyr81/94$^{2.58}$ (green line).

REFERENCES

[1] G. J. Martyna, D. J. Tobias, M. L. Klein, Constant pressure molecular dynamics algorithms, *The Journal of Chemical Physics*, **1994**, *101*, 4177–4189.
[2] E. Lyman, C. Higgs, B. Kim, D. Lupyan, J. C. Shelley, R. Farid, G. A. Voth, A role for a specific cholesterol interaction in stabilizing the Apo configuration of the human A(2A) adenosine receptor, *Structure*, **2009**, *17*,1660-1668.
[3] S. Nosé, A unified formulation of the constant temperature molecular-dynamics methods, *Journal of Chemical Physics*, **1984**, *81*, 511–519.
[4] W. G. Hoover, Canonical dynamics: Equilibrium phase-space distributions, *Phys. Rev. A*., **1985**, *31*, 1695–1697.
